# Directional nature of the product–moment correlation coefficient and some consequences

**DOI:** 10.3389/fpsyg.2022.988660

**Published:** 2022-10-17

**Authors:** Jari Metsämuuronen

**Affiliations:** ^1^Finnish Education Evaluation Centre (FINEEC), Helsinki, Finland; ^2^Turku Research Institute for Learning Analytics, University of Turku, Turku, Finland

**Keywords:** product–moment correlation coefficient, coefficient eta, directional coefficient, eta squared, Goodman–Kruskal *G*, Somers *D*

## Abstract

Product–moment correlation coefficient (PMC) is usually taken as a symmetric measure of the association because it produces an equal estimate irrespective of how two variables in the analysis are declared. However, in case the other variable has or both have non-continuous scales and when the scales of the variables differ from each other, PMC is unambiguously a directional measure directed so that the variable with a wider scale (*X*) explains the order or response pattern in the variable with a narrower scale (*g*) and not in the opposite direction or symmetrically. If the scales of the variables differ from each other, PMC is also prone to give a radical underestimation of the association, that is, the estimates are deflated. Both phenomena have obvious consequences when it comes to interpreting and speaking of the results. Empirical evidence shows that the effect of directionality increases by the discrepancy of the number of categories of the variables of interest. In the measurement modelling setting, if the scale of the score variable is four times wider than the scale of the item, the directionality is notable: score explains the order in the item and no other way around nor symmetrically. This is regarded as a positive and logical direction from the test theory viewpoint. However, the estimate of association may be radically deflated, specifically, if the item has an extremely difficult level. Whenever the statistic *r*^2^ or *R*^2^ is used, as is usual in general scatterplots or when willing to express the explaining power of the variables, this statistic is always a directional measure, and the estimate is an underestimate if the scales differ from each other; this should be kept in mind when interpreting *r*-squared statistics as well as with the related statistic eta squared within general linear modelling.

## Introduction

The coefficients of the association are divided into symmetric and directional ones. Although the directionality itself is often defined loosely, the directional measures give (generally) two options for the association: one of the variables is dependent and the other is independent, whereas the symmetric measures handle both variables as independent ones. Then, traditionally, the symmetric measures produce only one estimate of the association. Some traditional directional measures of association are Goodman–Kruskal lambda and tau (Goodman and Kruskal, 1954), Somers delta (*D*; Somers, 1962), and coefficient eta (η; Pearson, 1903, 1905), and some with a symmetric nature are phi (Pearson, 1904) and Kendall tau-a and tau-b (Kendall, 1938, 1948).

Some coefficients, such as Goodman–Kruskal gamma gamma (*G*; Goodman and Kruskal, 1954) and product–moment correlation coefficient (PMC; Bravais, 1844^1^ ; Galton, 1889; Pearson, 1896; onward), are traditionally taken as symmetric measures (see e.g., IBM, 2017a) because they produce only one estimate for the association regardless of how two variables, e.g., *X* and *Y*, are declared; the outcome takes on the same value declared either way (e.g., Walk and Rupp, 2010). However, both *G* and PMC have, factually, a hidden directional nature (see, e.g., Metsämuuronen, 2021b,2022a), and this character related to PMC is the main interest in this article; this is discussed later. Although the phenomenon is general, the focus is on the educational settings and the datasets in the numerical examples come from datasets related to student assessment.

Metsämuuronen (2022b) noted that of the coefficients of association between two variables, PMC has the longest history and the widest applicability. This may be the reason why it is referred to in academic works more than the other coefficients taken together; at the time of finalising this article (October 2022), according to Google Scholar, PMC is referred to in more than 1.4 million publications (523,000 times as “product–moment”, 883,000 times as “Pearson correlation”, and 47,000 times as “point biserial”). For instance, the second most frequent family of coefficients, Kendall tau, including tau-a, tau-b, and tau-c, is referred to around 92,000 times (“Kendall tau” and “Kendall’s tau”); “biserial correlation” is referred to 32,000 times (including rank biserial and point biserial correlations); “Somers delta” is referred to 30,000 times; “polychoric correlation” is referred to 12,000 times; and coefficients by Goodman and Kruskal, including gamma, tau, and lambda, are referred to around 5,600 times (“Goodman–Kruskal”).

The wide applicability of PMC has led to its wide use in numerous applications. PMC is used in procedures related to factor analysis, structural equation modelling, and regression analysis; in item analysis as one of the classical estimators of the item–score association; and in estimating reliability. Metsämuuronen (2022b) pointed out that PMC has been, and it still is, one of the main engines of many practical applications in modern scientific inquiries and analysis settings. The wide use of PMC alone motivates us to study the hidden characteristics of PMC: the directional nature may necessitate to reconsider our interpretations related to the estimates. Pearson himself, however, valued the *polychoric* correlation coefficient (Pearson, 1900, 1913) generalised from tetrachoric correlation (Pearson, 1900) as his most important contribution to the research community [based on a note by Pearson’s colleague Burton H. Camp (1933); see discussion in Ekström (2011)].

Because of more than a century of research with and on PMC, some of its weaknesses or challenges are well known, and those are discussed in general textbooks (e.g., Salkind, 2010; Tabachnick and Fidell, 2013; Metsämuuronen, 2017). Three relevant matters from the viewpoint of the topic of this article are discussed in this study. First, PMC is not powerful when the phenomenon is curvilinear; the factual association may be perfect, but PMC cannot detect this. In such settings, η is more powerful in reflecting curvilinear association (e.g., Ayres, 1920; Sechrest and Yeaton, 2011; Howell, 2012). For the non-linear settings, the distance correlation (Székely et al., 2007) based on Euclidean distances instead of strict covariation may be the best option though [see a simple introduction in Gleeson (2018); see literature and fast algorithm in Chaudhuri and Hu (2019); see R codes in Gleeson, 2018; Rizzo and Székely, 2022].

Second, Meade (2010) and Walk and Rupp, (2010; see also Sackett and Yang, 2000; Sackett et al., 2007) remind us of the phenomenon of restriction of range, which is that when only a portion of the range of values of a variable is actualised in the sample, it leads to inaccurate estimates of correlation by PMC. This may happen, for example, when only the highest-achieving students from the population apply to a study programme, causing the variance in the entrance test to be reduced remarkably (see illustrations of different patterns of restriction of range in Sackett and Yang, 2000). This phenomenon is called attenuation, which, in general, refers to underestimating the correlation between two different measures because of measurement error (Silver, 2008). Pearson offered the first solution to correct the attenuation in 1903, and many solutions have been offered since (see the typology in Mendoza and Mumford, 1987; Sackett et al., 2007). This characteristic of PMC has been studied and corrected, specifically, in the validity and meta-analytic studies (see literature and practices in Schmidt and Hunter, 2003, 2015; Schmidt et al., 2008).

Third, even though we would not face the traditional condition of restriction of range, PMC is severely affected by several sources of systematic and mechanical underestimation in the estimates of correlation (see Metsämuuronen, 2021a,b); the estimates may be radically deflated caused by artificial systematic errors during the estimation (see the discussion of the terms of attenuation and deflation in, e.g., Chan, 2008; Silver, 2008; Gadermann et al., 2012; Metsämuuronen, 2022a). For example, it is known that the number of categories and the division of the observations in the variable with the narrower scale (“item difficulty” in the testing settings) influence strictly the magnitude of the estimates of PMC (e.g., Martin, 1973, 1978; Olsson, 1980; Metsämuuronen, 2022d). It is known that when the categories of the variables are not equal, PMC cannot reach the perfect 1 due to mechanical reasons (see Metsämuuronen, 2017; see algebraic proof in Supplementary Appendix 1). In real-life settings, the deflation may be 0.60–0.70 units of correlation (see Metsämuuronen, 2022a). This is discussed later in Section “Practical notes on the deflation in product–moment correlation coefficient and R^2^” with a numerical example.

Based on simulations (Metsämuuronen, 2021b,2022d), at least six general factors that affect strictly and cumulatively the deflation in the estimates by PMC can be highlighted: (1) discrepancy in scales in general, (2) “difficulty level” and variance in *g*, (3) the number of categories in *g*, (4) the number of categories in the *X*, (5) the number of tied cases in *X*, and (6) the distribution of the latent variable. In the measurement modelling settings, specifically, when the scale in an item is radically narrower than the scale in the score, the estimates by the PMC between the item and the score—including factor loadings because, after all, factor loadings are PMCs between items and score (see Yang, 2010)—are always attenuated or deflated. This is specifically seen in the items with an extremely difficult level.

One specific characteristic of the PMC is that it is strictly connected to a genuinely directional estimator, coefficient eta. If the other variable is dichotomous and the other metric is ordinal, interval, continuous, or pseudo-continuous, PMC equals a specific direction of coefficient eta (see, e.g., Metsämuuronen, 2022a,b). Hence, PMC is a truly directional measure, but this character and its possible effects are not widely known or studied in real-life datasets. This article studies the magnitude and effects of this hidden characteristic of PMC. It is to be seen that this characteristic has a notable effect on our interpretation of the generally known and widely used statistic and its derivative, explaining power estimated by the squared correlation *r*^2^ or squared multiple correlation *R*^2^. There are many abbreviations in the article. These are collected in Supplementary Appendix 4.

## Research questions and the course of study

Although we know the algebraic connection between PMC and η, two relevant questions are largely unanswered: How remarkable is the effect of directionality in PMC in real-life settings? and What are the consequences of the directionality?

The course of the study starts with a discussion of the different forms of PMC and its connection to coefficient eta in the dichotomous case in Section “Some characteristics of PMC and the connection with coefficient eta.” In this section, some further algebraic treatments of the directionality of PMC in the polytomous case are given and also a possible benchmarking estimator for the deflation in PMC, attenuation-corrected PMC, is discussed. Section “Numerical examples of the directional nature of product–moment correlation coefficient” offers numerical examples and small studies of the effect of directionality. The methodological decisions are discussed in Section “Datasets, methods, and the course of study.”

## Some characteristics of product–moment correlation coefficient and the connection with coefficient eta

### Different forms of product–moment correlation coefficient

Basically, PMC is a measure of observed association for two continuous variables (see a typology in Metsämuuronen, 2022c). In general, PMC is the standardised covariation between variables *X* and *Y*:


(1) $$\rho_{X\,Y}=\frac{\sigma_{X\,Y}}{\sigma_{X}\,\sigma_{Y}}$$

where *σ*_*XY*_ is the covariance and *σ*_*X*_ and *σ*_*Y*_ are standard deviations of *X* and *Y*, respectively. However, its computational mechanics is also used in such measures as point biserial correlation (*RPB*) between a binary variable and a metric variable (with an ordinal, interval, or continuous scale) and point polyserial correlation coefficient (*RPP*) between an ordinal variable with a narrower scale and a metric variable with a wider scale. The mechanics is also used in the rank correlation coefficient, famously simplified by Spearman (1904). Additionally, PMC is embedded in the standard procedures of estimating biserial, polyserial, and polychoric correlation coefficients for the inferred association between an observed binary or ordinal variable and a latent non-observable metric variable or between two non-observable latent variables (of the procedures, see Olsson et al., 1982; Drasgow, 1986).

If the other variable is a binary or dichotomous one, a simplified form for the point biserial correlation is found in the textbooks (e.g., Lord et al., 1968; Lane et al., 2016; Metsämuuronen, 2017):


(2) $$\text{PMC}=\rho_{P\,B}=\rho_{g\,X}=\left({\bar{X}}_{1}-{\bar{X}}_{0}\right)\times \frac{\sigma_{g}}{\sigma_{X}}$$

originally provided by Swineford (1936) and Kuder (1937),^2^ where ${\bar{X}}_{0}$ and ${\bar{X}}_{1}$ refer to the means of the variable *X* in the subpopulations 0 and 1 of *g* and $\sigma_{g}=\sqrt{\left(n_{0}\times n_{1}\right)\,/\,{\left(n_{0}+n_{1}\right)}^{2}}=\sqrt{p\,\left(1-p\right)}$ and *σ*_*X*_ are standard deviations of *g* and *X*, respectively. Eq. 2 appears to be important in understanding the directional nature of PMC. Namely, Wherry and Taylor (1946) and Eikeland (1971) showed that a certain direction of a truly directional coefficient eta equals Eq. 2. A simplified proof is given in Supplementary Appendix 2.

### Directionality in the dichotomous case

Of the directional estimators of association, coefficient eta, sometimes called the correlation ratio, specifically, in the early days (chronologically, e.g., Pearson, 1911; Ayres, 1920; Fisher, 1925; Kelley, 1935), appears to be an interesting coefficient from the viewpoint of focus in this article because it has a strict connection to PMC. Coefficient η can be expressed in multiple ways. Here, the form familiar from the settings related to general linear modeling (GLM) is used.

Assume two variables *g* and *X* with observed values *x* and *y*, respectively, with *x* = 1, …, *R* and *y* = 1, …, *C* categories and *R* < < *C*. Although η is often used in estimating the association between a nominal-scaled variable and a metric variable, its computational mechanism can be used, obviously, with ordinal or even interval-scaled variables. In many cases related to η, the widths of the scales are far from each other (*R* < < *C*) although this is not a necessity in calculating Euclidian. The condition of *R* < < *C* is specifically true when we consider a binary or dichotomous *g* by a metric *X*.

In general, the traditional direction of η directed so that “*X* dependent” (usually η(*X*|*g*) in the settings related to GLM) or “*g* given *X*” (usually η(*g*|*X*) or, here, η_*g*|*X*_ in the settings related to conditions)^3^ can be expressed as follows:


(3) $$\eta_{g|X}=\sqrt{\eta_{g|X}^{2}}=\sqrt{\frac{S\,S_{b\,e\,t\,w\,e\,e\,n}\,\left(g|X\right)}{S\,S_{t\,o\,t\,a\,l}\,\left(g|X\right)}}$$

$$=\sqrt{\frac{\sum_{g=1}^{R}n_{g}\,{\left({\bar{X}}_{g}-G\,M_{X}\right)}^{2}}{\sum_{i=1}^{N}{\left(y_{i}-G\,M_{X}\right)}^{2}}},$$

where ${\bar{X}}_{g}$ refers to the means of *X* in the subpopulations in *g*, and *GM*_*X*_ is the grand mean of *X*. The opposite direction “*g* dependent” or “*X* given *g*” can be expressed as follows:


(4) $$\eta_{X|g}=\sqrt{\eta_{X|g}^{2}}=\sqrt{\frac{S\,S_{b\,e\,t\,w\,e\,e\,n}\,\left(X|g\right)}{S\,S_{t\,o\,t\,a\,l}\,\left(X|g\right)}}$$

$$=\sqrt{\frac{\sum_{X=1}^{C}n_{X}\,{\left({\bar{g}}_{X}-G\,M_{g}\right)}^{2}}{\sum_{i=1}^{N}{\left(x_{i}-G\,M_{g}\right)}^{2}}},$$

where ${\bar{g}}_{X}$ refers to the mean of *g* in each category *X*, and *GM*_*g*_ is the grand mean of *g*. Except in the special case where the variables have equal scales with no crossing categories, η_*g*|*X*_ ≠η_*X*|*g*_. If we obtain, in a specific dataset, values η_*g*|*X*_ = 0.7595 and η_*X*|*g*_ = 1, in the framework of GLM, we would infer that *g* explains 59% (= 0.76^2^) of the variability in *X* while *X* explains the variability in *g* perfectly (100%). Within measurement modelling settings, the interpretation is opposite: *X* explains 59% of the response pattern in *g* and the latter direction is not meaningful (see footnote 3). However, in general, both directions η_*g*|*X*_ and η_*X*|*g*_ may make sense in some practical settings; for example, we may be interested in whether the attitude explains more the achievement or is it the opposite.

In the binary case, Eq. 3 is reduced to


(5) $$\eta_{g|X}=\sqrt{\frac{n_{0}\,{\left({\bar{X}}_{0}-G\,M_{X}\right)}^{2}+n_{1}\,{\left({\bar{X}}_{1}-G\,M_{X}\right)}^{2}}{\sum_{i=1}^{N}{\left(y_{i}-G\,M_{X}\right)}^{2}}},$$

where ${\bar{X}}_{0}$ and ${\bar{X}}_{1}$ are the means of *X* in the subpopulations of *g* = 0 and 1. This form appears to be important when showing the directional nature of PMC. Independently, Wherry and Taylor (1946) and Eikeland (1971) showed that, in the binary case, Eq. 5 can be expressed in the following form:


(6) $$\eta_{g|X}=\left({\bar{X}}_{1}-{\bar{X}}_{0}\right)\,\frac{\sigma_{g}}{\sigma_{X}}\neq \eta_{X|g}$$

which, obviously, equals the simplified form of point biserial correlation in Eq. 2. Then, with binary items or, for example, with dummy variables in GLM settings (see Cohen, 1969), PMC equals η directed so that “*g* given *X”* (in the measurement modelling settings) or “*X* dependent” (in GLM settings) but not with the opposite direction of eta:


(7) $$\mathrm{PMC}=R_{P\,B}=\rho_{g\,X}=\eta_{g|X}\neq \eta_{X|g}.$$

This means that PMC is, factually, a directional measure in the binary case. In what follows, some algebraic evidence of the phenomenon in the polytomous cases is discussed.

### Directionality in the polytomous case

In the polytomous case, PMC and η_*g*|*X*_ differ from each other by a small but important thing. We remember (see, e.g., Howell, 2012) that by using the concepts of sums of squares, the absolute value of PMC can be expressed as follows:


(8) $$\left|\text{PMC}\right|=\sqrt{\frac{S\,S_{t\,o\,t\,a\,l}-S\,S_{r\,e\,s\,i\,d\,u\,a\,l}}{S\,S_{t\,o\,t\,a\,l}}}$$

and the absolute value of η as follows:


(9) $$\left|\eta_{g|X}\right|=\sqrt{\frac{S\,S_{b\,e\,t\,w\,e\,e\,n}}{S\,S_{t\,o\,t\,a\,l}}=}\,\sqrt{\frac{S\,S_{t\,o\,t\,a\,l}-S\,S_{e\,r\,r\,o\,r}}{S\,S_{t\,o\,t\,a\,l}}}.$$

Unconventionally, η is expressed with absolute signs in this study. The value of η as we usually see it is, in fact, the *absolute value* of the association between two variables—this is discussed later in Section “Some related consequences of the relationship of product–moment correlation coefficient and eta.” Hence, after simplifying, we can express the difference between PMC and η as follows:


(10) $$\left|\text{PMC}\right|=\sqrt{\frac{\sum {\left(X_{i\,j}-\bar{X}\right)}^{2}-\sum {\left(X_{i\,j}-{\hat{X}}_{i\,j}\right)}^{2}}{\sum {\left(X_{i\,j}-\bar{X}\right)}^{2}}},$$

and


(11) $$\left|\eta_{g|X}\right|=\sqrt{\eta_{g|X}^{2}}=\sqrt{\frac{\sum {\left(X_{i\,j}-\bar{X}\right)}^{2}-\sum {\left(X_{i\,j}-{\bar{X}}_{j}\right)}^{2}}{\sum {\left(X_{i\,j}-\bar{X}\right)}^{2}}}.$$

Only in the specific case the association between two variables is perfectly linear, the predicted value by the regression model (${\hat{X}}_{i\,j}$) equals the means of the subpopulations in *g* (${\bar{X}}_{j}$). In this specific case, which is always true in the binary and dichotomous cases and may occur, although rarely, in the polytomous case, $\sum {\left(X_{i\,j}-{\hat{X}}_{i\,j}\right)}^{2}$ = $\sum {\left(X_{i\,j}-{\bar{X}}_{j}\right)}^{2}$and, consequently, |PMC| = |η_*g*|*X*_|. In any other condition, $\sum {\left(X_{i\,j}-{\hat{X}}_{i\,j}\right)}^{2}$ < $\sum {\left(X_{i\,j}-{\bar{X}}_{j}\right)}^{2}$ because $\sum {\left(X_{i\,j}-{\hat{X}}_{i\,j}\right)}^{2}$ is constructed so that the residuals related to the data in hand would be the *minimum* and, hence, $\sum {\left(X_{i\,j}-{\bar{X}}_{j}\right)}^{2}$ would always be greater than the minimum, causing:


(12) $$\left|\text{PMC}\right|\leq \left|\eta_{g|X}\right|.$$

Hence, except for the case of linearity in (ordinal) *g*, which is a rare case in general but always the condition in binary and dichotomous settings, the absolute magnitude of the estimates by PMC is always lower than that by η_*g*|*X*_. Obviously, the comparison makes sense only with categorical *ordinal g*; PMC does not get meaningful interpretations with categorical nominal *g*.

### Some related consequences of the relationship of product–moment correlation coefficient and eta

The algebraic connection between PMC and η has some consequences not only in PMC (the directionality) but also in interpreting η and η^2^ discussed by Metsämuuronen (2022a). First, the traditional way of calculating η (by taking the second powers of all elements in the formula) will lead to an apparent positive association. However, in fact, η_*g*|*X*_ can also reach negative values: if in Eq. (6), ${\bar{X}}_{1}<{\bar{X}}_{0}$, the factual correlation is negative. Hence, η calculated by using the traditional formulae gives us only the *magnitude* of the association and not necessarily the true association.

Second, the connection of PMC and η_*g*|*X*_ makes it clear why the *estimates η_*g*|*X*_ underestimate the true association in an obvious manner*. The underestimation in η_*g*|*X*_ is mechanical in nature in the same manner as in PMC discussed above. The magnitude of this obvious underestimation in PMC, and, consequently, in η_*g*|*X*_ depends on several factors; six of those were given above, and Metsämuuronen (2022d) discusses 11 such sources. In the extreme binary case, when the proportion of 1s (*p*) or 0s (1–*p*) approximates *p* = 1 or *p* = 0, η_*g*|*X*_ approximate zero regardless of the true association between the variables. This is seen when comparing the magnitude of the estimates by PMC and η with ones obtained by such estimators that *can* detect the perfect association in extreme datasets (e.g., *RPC*, *G* or *D*, see Metsämuuronen (2021b,2022e,f). The phenomenon also generalises to polytomous cases. Some examples of the magnitude of the mechanical underestimation are illustrated with numerical examples in Section “Numerical examples of the directional nature of product–moment correlation coefficient.”

Third, because the estimates by η_*g*|*X*_ are deflated in an obvious manner, also $\eta_{g|X}^{2}$ must underestimate the true explaining power in an obvious manner. Inherited from η_*g*|*X*_, the magnitude of the obvious underestimation in $\eta_{g|X}^{2}$ depends on several factors, such as the number of categories in *g* and *X*, the proportion of 0 s and 1 s (or the variance) in *g*, and the distribution of the latent variable (see more factors in Metsämuuronen, 2022d). In an extreme dichotomous case, $\eta_{g|X}^{2}$ approximates zero irrespective of the true association between the variables if $\sigma_{g}^{2}$ approximates 0. Then, factually, latent to a set of an extremely unbalanced dichotomous variable (e.g., consisting only of a few cases of males or females or with items of extremely difficult level) and a metric variable (e.g., score), there could be a perfect correlation, which should be detected as perfect explaining power. However, $\eta_{g|X}^{2}$ cannot detect this because it cannot reach the limits of correlation due to deflation, and the deflation may be remarkable, 50–70% or even more (see examples in Metsämuuronen, 2022a).

Metsämuuronen (2022a) notes that the obvious underestimation by $\eta_{g|X}^{2}$ evokes justified questions related to the traditional corrections connected with $\eta_{g|X}^{2}$. The biased-corrected measures such as omega squared (ω^2^; Hays, 1963), epsilon squared (ε^2^, Kelley, 1935), and adjusted eta squared ($\eta_{a\,d\,j}^{2}$; Mordkoff, 2019) are developed to correct the *positive bias* in η^2^, specifically, with values near zero (see Okada, 2013, 2017; Mordkoff, 2019). These measures tend to make the value of η^2^ even more deflated. For this, Metsämuuronen (2022a,e) suggests an attenuation correction for point biserial and point polyserial correlation coefficients as well as for η_*g*|*X*_ and $\eta_{g|X}^{2}$. These are briefly discussed as follows.

### Practical notes on the deflation in product–moment correlation coefficient and R^2^

Above, it was noted that one of the deficiencies of PMC is its tendency to produce deflated estimates caused by technical or mechanical error in the estimation. This can be illustrated by taking a pair of identical variables with an obvious perfect correlation (*ρ*_*θθ*_ = 1). This setting corresponds with the latent image of the measurement model with one latent variable θ (such as “achievement in mathematics”) which is manifested as the score in an item with a narrower scale and in a score variable with a wider scale.

**FIGURE 1:**
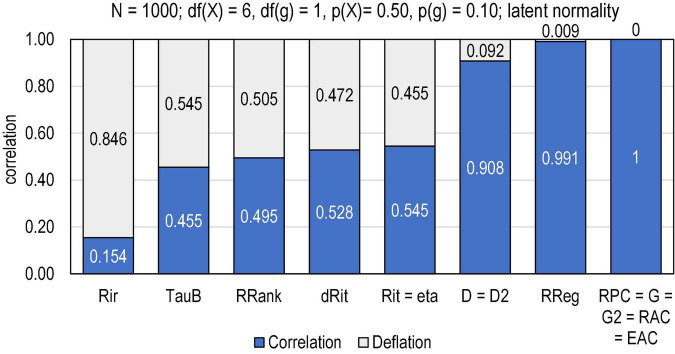
Magnitude of deflation in the estimates by selected estimators of association. Rir = Henrysson item–rest correlation (= PMC);Tau-b = Kendall tau-b; RRank = Spearman rank-order correlation (= PMC); dRit = distance correlation; Rit = item–score correlation (= PMC); eta = coefficient eta (X dependent) (= PMC in the binary case); D = Somers delta (X dependent); D2 = dimension-corrected D; RReg = r-bireg correlation; RPC = polychoric correlation; G = Goodman–Kruskal gamma; G2 = dimension-corrected G, RAC = attenuation-corrected Rit; EAC = attenuation-corrected eta.

Let us take a vector of *n* = 1,000 normally distributed cases and double it. Of these (identical) variables, one (score *X*) is divided into 7 categories (*df*(*X*) = 6) with the difficulty level of *p*(*X*) = 0.50, and the other (item *g*) is divided into a binary form (*df*(*g*) = 1) by using a cut-off of *p*(*g*) = 0.10, that is, 90% of the hypothetical test-takers gave the incorrect answer. This could be a latent reflection of a very difficult item in a subtest (e.g., “sets”) amidst a longer test (“achievement in mathematical”). The difference between the observed item–score correlation (*Rit* = *ρ*_*iX*_) and the latent correlation (*ρ*_*θθ*_ = 1) indicates strictly the magnitude of deflation in the estimate; in Figure 1, relevant benchmarking estimators of the association are compared in this regards.

From the deflation viewpoint, it is notably that such estimators of item–score association based on the mechanics of PMC as Henrysson’s item–rest correlation *Rir* (Henrysson, 1963), Spearman rank-order correlation *R*_*Rank*_ (Spearman, 1904), *Rit*, and η, cannot detect the true perfect latent correlation, and the magnitude of deflation is notable (>0.45 units of correlation). Also, Kendall *tau-b* (Kendall, 1948) gives a notably deflated estimate—the magnitude of its estimates is always lower than those by PMC (see, e.g., Metsämuuronen, 2021b)—as well as the correlation based on distance instead of covariance and distance correlation (*dRit*; Székely et al., 2007).^4^ In contrast, such estimators as polychoric correlation (*R*_*PC*_; Pearson, 1900, 1913), Goodman–Kruskal gamma (*G*; Goodman and Kruskal, 1954), dimension-corrected *G* (*G*_2_; Metsämuuronen, 2021a), and attenuation-corrected *Rit* and *eta* (*R*_*AC*_ and *E*_*AC*_; Metsämuuronen, 2022a,e) *can* detect the latent perfect correlation. Such estimators as r-bireg correlation (*R*_*REG*_; Livingston and Dorans, 2004; Moses, 2017), Somers delta directed so that “score dependent” (*D*; Somers, 1962) and dimension-corrected *D* (*D*_2_; Metsämuuronen, 2020b,2021a) come close the deflation-free outcome. Obviously, when PMC (= *r*) is deflated, also *r*^2^ is deflated and, hence, the explaining power may be radically deflated. In the case related to Figure 1, *Rit* = 0.545 leads to *Rit*^2^ = 0.297, that is, even if the true latent explaining power between the variables is perfectly $\rho_{\theta \,\theta }^{2}=1$, only 29,7% of this can be reached when used PMC in the estimation.

When it comes to the multiple correlation coefficient (*R*) and squared multiple correlation (*R*^2^) used widely with the regression models for the explaining power of multiple independent variables [see other options in Kvålseth (1985) and expanded in Onyutha (2022)], the deflation in PMC is also inherited to these statistics. Let us assume the same hypothetical settings as above with perfect correlations between variables, but now we have one dependent variable (score *X*) with *p* = 0.50 and latent normality and two «independent» different manifestations of the same original variable (items *g*_1_ and *g*_2_) explaining the score. Let the items be binary with the difficulty levels *p*(*g*_1_) = 0.2 and *p*(*g*_2_) = 0.80. Keeping in mind that the latent images of these three variables are identical, the observed correlations as follows:

**Table:** 

	*X*	*g*_1_ (*p* = 0.20)	*g*_2_ (*p* = 0.80)
*X*	1	0.66055	0.66055
*g*_1_ (*p* = 0.20)		1	0.25313
*g*_2_ (*p* = 0.80)			1

In the case of two independent variables, *R* is computed by the formula $R_{y.x\,z}=\sqrt{\frac{\rho_{y\,x}^{2}+\rho_{y\,z}^{2}-2\,\rho_{y\,x}\,\rho_{y\,z}\,\rho_{x\,z}}{1-\rho_{x\,z}^{2}}}$, where *y* refers to the dependent variable, and *x* and *z* are the independent variables. Here, *y* = *X*, *x* = *g*_1_, and *z* = *g*_2_. The estimate for the multiple correlation coefficient is $R=\sqrt{\left(2\times 0.661^{2}-2\times 0.661\times 0.661\times 0.253\right)\,/\,\left(1-0.253^{2}\right)}$ = 0.834 and, consequently, *R*^2^ = 0.696, that is, less than 70% of the phenomenon was explained.

### Attenuation-corrected product–moment correlation coefficient and attenuation-corrected eta

Because of the attenuation and deflation in the estimates by PMC, Metsämuuronen (2022e) proposed a simple correction of attenuation for PMC. The correction is based on the fact that, given the dataset, the correlation between two variables cannot exceed the limit specified by the observed values in these variables—the same logic can be used also with the attenuation-corrected eta (Metsämuuronen, 2022a). Namely, when the observed values in two variables are given, the variances ($\sigma_{X}^{2}$ and $\sigma_{g}^{2}$) are fixed. Recalling the basic formula of PMC (ρ_*gX*_ = σ_*gX*_/σ_*g*_σ_*X*_), the only element affecting the magnitude of the correlation is the covariance between the variables (*σ*_*gX*_). The maximum value of *σ*_*gX*_ ($\sigma_{g\,X}^{M\,a\,x}$) is obtained when *g* and *X* are in the *same order*. Hence, the maximal possible correlation ($\rho_{g\,X}^{M\,a\,x}$) in the given set of variables is given as follows (see Metsämuuronen, 2022e):


(13) $$\rho_{g\,X}^{M\,a\,x}=\frac{\sigma_{g\,X}^{M\,a\,x}}{\sigma_{g}\,\sigma_{X}}.$$

Based on this, the attenuation-corrected PMC (*ρ*_*AC*_, *R*_*AC*_) is the proportion of the observed correlation ($\rho_{g\,X}^{O\,b\,s}$) of the maximal possible correlation ($\rho_{g\,X}^{M\,a\,x}$) given the observed values in the variables:


(14) $$\rho_{A\,C}=\frac{\rho_{g\,X}^{O\,b\,s}}{\rho_{g\,X}^{M\,a\,x}}.$$

In the same manner, the attenuation-corrected η_*g*|*X*_ (η_*AC*_, *E*_*AC*_) is the proportion of observed eta ($\eta_{g|X}^{O\,b\,s}$) to the maximal eta in the given dataset ($\eta_{g|X}^{M\,a\,x}$), which, in the binary case, equals the maximum value of PMC:


(15) $$\eta_{A\,C}=\frac{\eta_{g|X}^{O\,b\,s}}{\eta_{g|X}^{M\,a\,x}}.$$

In the case of η_*g*|*X*_, as with PMC, the maximal η_*g*|*X*_ is found when the variables are ordered independently and η_*g*|*X*_ is calculated between these variables (see Metsämuuronen, 2022a). Consequently, the attenuation-corrected eta squared is the square of *E*_*AC*_, that is, $E_{A\,C}^{2}$.

The characteristics of *R*_*AC*_ and *E*_*AC*_ are studied by Metsämuuronen (2022d). It was noticed that they were free of deflation in 9 out of 11 sources of deflation and, hence, they were ranked high in the set of estimators of correlation when it comes to deflation-free characteristics of different estimators. Therefore, Metsämuuronen (2022f) suggests these to be used in deflation-corrected estimators of reliability.

## Numerical examples of the directional nature of product–moment correlation coefficient

### Datasets, methods, and the course of study

The empirical section studies different aspects of directionality in PMC. First, Section “Directionality in the binary and polytomous datasets—a simple comparison” illustrates the directionality in binary and polytomous ordinal cases by using a simple comparison of different patterns in the variable with a narrower scale. The characteristics of a simple, hypothetical dataset with purposeful patterns are discussed in detail in that section. In this section, no specific statistical methods are used.

Second, the magnitude of the directionality in real-life settings is studied in Sections “Directionality of product–moment correlation coefficient in the binary and polytomous cases in real-life datasets” and “How remarkable is the effect of directionality in product–moment correlation coefficient?” from two perspectives. Section “Directionality of product–moment correlation coefficient in the binary and polytomous cases in real-life datasets” studies the magnitude of the directionality in general, and Section “How remarkable is the effect of directionality in product–moment correlation coefficient?” studies how remarkable the effect of directionality in PMC is. The derivation and basic results mentioned above are general and applicable without connection to any specific framework. However, the numerical examples and the applications in Sections “Directionality of product–moment correlation coefficient in the binary and polytomous cases in real-life datasets,” “How remarkable is the effect of directionality in product–moment correlation coefficient?,” “Relation of product–moment correlation coefficient and R_*AC*_” are discussed within the measurement modelling settings and, specifically, in the item analysis settings where the point biserial and point polyserial correlation coefficients (*ρ*_*gX*_), that is, item–score correlation (*Rit* = PMC) is of interest.

In Sections “Directionality of product–moment correlation coefficient in the binary and polytomous cases in real-life datasets” and “How remarkable is the effect of directionality in product–moment correlation coefficient?,” the relation of the estimates by PMC and η is studied by using two larger real-world datasets, training dataset and cross-validating dataset, related to item analysis of the test scores of mathematics achievement (see Supplementary Appendix 3 of forming of the datasets). In the original dataset of 4,023 test takers of a mathematics test with 30 binary items (FINEEC, 2018), the item–score correlation varied 0.332 < ρ_*gX*_ < 0.627 with the average $\bar{\rho_{g\,X}}=0.481$, the difficulty levels of the items varied 0.24 < *p* < 0.95 with the average $\bar{p}$ = 0.63, and the lower bound of reliability was α = 0.885. For the training dataset, a set of 1,080 real-world tests were produced with a different number of test-takers (*n* = 50, 100, and 200), number of items (*k* = 2–30, $\bar{k}=10.22$), difficulty levels ($\bar{p}$ = 0.55–0.76, $\bar{\bar{p}}=0.661$), reliabilities (α = 0.739–0.935, $\bar{\alpha }=0.862$), and degrees of freedom in the item (*df*(*g*) = *R*–1 = 1–15, $\bar{d\,f\,\left(g\right)}=5.06$) and in the score (*df*(*X*) = *C*–1 = 12–27, $\bar{d\,f\,\left(X\right)}=19.2$). These tests produced 11,160 test items with varying difficulty levels, item variances, number of categories, as well as estimates by PMC and η. The dataset is available at http://dx.doi.org/10.13140/RG.2.2.24238.02889. Notably, in Section ‘‘Relation of product--moment correlation coefficient and R_*AC*_,’’ a somewhat larger dataset is used; this dataset includes also a sample size of *n* = 25.^5^ The smaller dataset is used in this study mainly because of the possibility to compare the results with a larger cross-validating dataset, which was produced by using only sample sizes of *n* ≥ 50. For the cross-validating dataset, the original 30 items were doubled with small changes in the order and response patterns of the real test-takers, and 29,887 items were produced. This had a small effect on the item difficulties and item–total correlations. The original items and the modified ones were combined as a dataset with 60 binary items with a nature of odd–even parallel tests. The latter dataset is referred to if the results between the datasets differ radically from each other.

Section “Directionality of product–moment correlation coefficient in the binary and polytomous cases in real-life datasets” does not use a specific statistical method; visual tools are used to illustrate the magnitude of the directionality. Section “How remarkable is the effect of directionality in product–moment correlation coefficient?” studies the magnitude of the directionality primarily by using the standard general linear modeling (GLM). Standard statistics such as eta squared (η^2^) and Cohen’s *f* and *d* (Cohen, 1988) are used to indicate the explaining power and effect sizes. The latter are used in assessing the practical effect of the directionality; for these, we have commonly accepted, rough boundaries for the low effect size (*d* < 0.20, *f* < 0.10) indicating only small effect, medium effect size (*d* ≈ 0.40, *f* = 0.20–0.30) indicating remarkable effect, and high effect size (*d* > 0.80, *f* > 0.40) indicating very remarkable effect. The *post hoc* tests are done by using Šidák’s routine (Šidák, 1967). Another statistical method, a data mining tool decision tree analysis (DTA; IBM, 2017b) with the algorithm CHAID (Chi-square Automatic Interaction Detector; Kass, 1980) is used in exploring the threshold cut-offs of interesting variables. This is used, for example, when willing to find cut-offs for the difference in the scales of two variables to indicate remarkable directionality. DTA is discussed later with more details.

Third, Section “Relation of product–moment correlation coefficient and R_*AC*_” studies the relation between PMC and attenuation-corrected PMC (*R*_*AC*_) suggested by Metsämuuronen (2022e). In this section, an enlarged dataset of 14,880 with a sample size of *n* = 25 is used (see text footnote 5). Paired-samples *t*-test and Cohen’s *d* are used to study the difference between PMC and *R*_*AC*_ on the one hand, and coefficient eta and *R*_*AC*_ on the other hand.

DTA may be a less known methodological tool for the average reader and, hence, it is further described in what follows. More information is found in the manual (IBM, 2017b) or in, e.g., Metsämuuronen (2017). DTA is a set of algorithms that detect cut-offs for categorising the explaining variables in groups where the discrimination is the most statistically significant. It goes through all possible combinations of categories and provides us with the one with the highest statistical significance. The decision is done by using Chi-Squared test (categorical dependent variable) or *F*-test (continuous dependent variable).

DTA is strong in finding such cut-offs for continuous (or categorical) explanatory variables that are practically unreachable by traditional analytical- or graphical methods (Metsämuuronen, 2017). In exploring two continues variables and their possible cut-offs as is the case in this article, it is difficult to find a more effective tool than these algorithms. Data mining is done by using different algorithms such as CHAID (Kass, 1980), Exhaustive CHAID (Biggs et al., 1991), CRT or CART, (Classification and Regression Trees; Breiman et al., 1984), and QUEST (Quick, Unbiased, *Efficient Statistical Tree*; Loh and Shih, 1997). The technical challenge in the algorithms—or factually in our processes of statistical inference—is that the algorithms tend to find the best solution when the unit sizes in the groups are high; it tends to combine the groups with small sample sizes. In the analysis to come in Section “How remarkable is the effect of directionality in product–moment correlation coefficient?”, the procedure is enhanced by doing further analysis in the extreme groups with smaller unit sizes without the middle part in most of the cases (see closer Table 5 and related discussion).

**TABLE 1:** Hypothetic example of the estimates by PMC and eta under different conditions.

	Rows (*g*_*i*_)						Column
Test taker	A1	A2	A3	B1	B2	B3	*X* (score)
1	0	0	0	0	0	0	1
2	0	0	0	0	0	0	2
3	0	0	0	0	0	0	3
4	0	0	0	0	0	0	4
5	0	0	0	0	1	0	5
6	0	0	1	0	0	1	6
7	0	0	0	0	0	0	6
8	0	0	0	0	0	0	6
9	0	0	1	0	0	0	6
10	0	0	0	0	0	0	10
11	0	0	0	0	0	0	11
12	0	0	1	0	1	1	12
13	0	0	0	0	0	0	13
14	0	1	0	0	0	0	14
15	0	0	1	1	2	2	15
16	1	0	0	1	0	0	16
17	1	1	0	1	0	0	17
18	1	1	1	2	1	1	18
19	1	1	0	3	3	3	19
20	1	1	0	4	4	4	20
PMC = Rit	0.759	0.721	0.117	0.747	0.603	0.610	
η1 “g given X”	0.759	0.721	0.117	0.820	0.606	0.614	
η2 “X given g”	1	1	0.856	1	1	0.985	
D “g given X”	1	0.947	0.200	1	0.646	0.292	
D “symmetric”	0.579	0.548	0.116	0.686	0.443	0.479	
D “X given g”	0.408	0.386	0.082	0.522	0.337	0.152	
G “g given X”	1	0.947	0.211	1	0.646	0.304	

**TABLE 2:** Selected characteristics of 11,160 items in comparison.

Range in the item scale	df(g)	Number of items	Average rit	Average η1^1^	Average η2^2^	Average item difficulty ($\bar{p}$)	Average item variance
0–1	1	5,943	0.486	0.486	0.646	0.661	0.210
0–2	2	2,265	0.625	0.632	0.730	0.664	0.503
0–3	3	1,029	0.708	0.719	0.788	0.668	0.883
0–4	4	546	0.771	0.782	0.828	0.666	1.373
0–5	5	354	0.812	0.823	0.861	0.647	2.027
0–6	6	278	0.846	0.857	0.885	0.675	2.738
0–7	7	190	0.873	0.882	0.899	0.661	3.635
0–8	8	98	0.892	0.903	0.918	0.686	4.688
0–9	9	127	0.911	0.921	0.933	0.655	5.913
0–10	10	118	0.926	0.934	0.941	0.643	7.153
0–11	11	85	0.940	0.948	0.954	0.673	8.784
0–12	12	61	0.943	0.951	0.956	0.682	10.232
0–15	13–14	66	0.944	0.950	0.956	0.667	11.649
Total		11,160	0.596	0.600	0.716	0.663	0.932

^1^η_*g*|*X*_ = eta directed so that “*g* given X” (as conditions) or “*X* dependent” (as in GLM).

^2^η_*X*|*g*_ = eta directed so that “*X* given *g*” (as conditions) or “*g* dependent” (as in GLM).

**TABLE 3:** Difference between eta1 and eta2 by df(g).

	Training dataset			Cross-validating dataset		
df(g)	Mean η2 – η1	Std. deviation	*N*	Mean η2 – η1	Std. deviation	*N*
1	0.15988	0.096480	5,943	0.14528	0.053269	20,426
2	0.09789	0.062310	2,265	0.10998	0.038545	3,898
3	0.06939	0.045693	1,029	0.08786	0.030073	1,253
4	0.04596	0.032817	546	0.05772	0.020795	1,131
5	0.03753	0.027430	354	0.05658	0.021637	1,830
6	0.02751	0.018152	278	0.06041	0.016985	133
7	0.01732	0.014543	190	0.02308	0.012072	13
8	0.01489	0.012894	98	0.02566	0.011268	229
9	0.01204	0.011770	127	0.02586	0.012140	488
10	0.00731	0.007255	118	0.02615	0.011412	230
11	0.00599	0.005562	85	0.01887	0.012374	47
12	0.00536	0.004957	61	0.0141	0.007802	101
13–14	0.00527	0.006326	66	0.01431	0.008364	108
Total	0.11628	0.093203	11,160	0.12419	0.059041	29,887

**TABLE 4:** Difference between eta1 and eta2 by C/R.

	Training dataset			Cross-validating dataset		
*C*/*R*^1^	Mean η2 – η1	Std. deviation	*N*	Mean η2 – η1	Std. deviation	*N*
≤1.929	0.00662	0.00714	363	–	–	–
(1.929–2.500]	0.01715	0.01538	273	0.01117	0.005443	130
(2.500–3.111]	0.02683	0.022606	348	0.01907	0.009835	402
(3.111–3.909]	0.04267	0.033194	524	0.02754	0.011277	562
(3.909–4.333]	0.05552	0.043617	341	0.03413	0.011424	106
(4.333–5.000]	0.06759	0.05038	563	0.04332	0.017637	493
(5.000–6.833]	0.09558	0.066038	1,587	0.05914	0.020941	2,375
(6.833–9.333]	0.15063	0.104120	2,541	0.07974	0.027926	1,293
(9.333–10.50]	0.15586	0.099859	2,369	0.10105	0.036171	2,510
(10.50–12.50]	0.13654	0.077862	1,923	0.1152	0.040712	5,897
(12.50–14.00]	0.10871	0.050848	328	0.13006	0.043605	4,388
(14.00–15.50]	–	–	–	0.14309	0.04652	3,650
(15.50–16.50]	–	–	–	0.15407	0.048539	1,895
(16.50–18.00]	–	–	–	0.17029	0.049647	2,540
(18.00–19.00]	–	–	–	0.17869	0.053302	1,922
(19.00–20.00]	–	–	–	0.18588	0.054029	964
>20.00	–	–	–	0.19155	0.055442	760
Total	0.11628	0.093203	11,160	0.12419	0.059041	29,887

**Statistics related to the solutions**						

F; (Sig.)	356.03; (*p* < 0.001)			1,927.03; (*p* < 0.001)		
Eta squared	0.242			0.492		
Cohen’s f	0.565			0.984		

^1^*C* = number of categories in *X*, *R* = number of categories in *g*.

**TABLE 5:** Relation of PMC and RAC.

	Correlations				Number of cases	Standard deviations			
df(g)	RgX	RAC	Eta1	Eta2	*n*	RgX	RAC	Eta1	Eta2
1	0.473	0.586	0.473	0.682	7,948	0.136	0.166	0.118	0.136
2	0.612	0.675	0.625	0.755	3,056	0.110	0.117	0.092	0.104
3	0.701	0.746	0.717	0.807	1,390	0.085	0.088	0.072	0.081
4	0.766	0.801	0.784	0.843	729	0.071	0.071	0.058	0.065
5	0.809	0.838	0.828	0.873	474	0.058	0.058	0.049	0.054
6	0.848	0.871	0.865	0.897	366	0.037	0.038	0.039	0.038
7	0.878	0.897	0.893	0.912	255	0.032	0.034	0.035	0.035
8	0.901	0.918	0.916	0.933	140	0.028	0.029	0.031	0.030
9	0.916	0.931	0.929	0.940	160	0.026	0.027	0.026	0.028
10	0.929	0.942	0.939	0.947	136	0.020	0.022	0.023	0.023
11	0.941	0.951	0.950	0.956	93	0.013	0.014	0.017	0.017
12	0.945	0.955	0.954	0.959	67	0.011	0.013	0.014	0.014
13	0.944	0.952	0.950	0.957	42	0.008	0.008	0.011	0.009
14	0.945	0.953	0.951	0.955	24	0.008	0.008	0.009	0.008
Total	0.584	0.666	0.591	0.744	14,880	0.182	0.172	0.128	0.186

### Directionality in the binary and polytomous datasets—a simple comparison

The estimates by PMC and η are compared first by using a simple dataset comprising 6 variables with a narrower scale (*g*) and a common metric variable (*X*) with a wider scale (Table 1). This could be a partial dataset from an achievement testing with six selected items *g*_*i*_ and the score *X*. From now on, to shorten the expression, η1 refers to η(*g*|*X*) or “*g* given *X*” or “*X* dependent” in the outputs of software packages, which are the relevant direction from both GLM and measurement modelling viewpoint, and η2 refers to η(*X*|*g*) or “*X* given *g*” or “*g* dependent” in the outputs of software packages, which may be a relevant direction in the general case. Because η, *G*, and *D* are usually calculated from a form of cross-table, variable *g* is labelled to be in “Row” and *X* in “Column.” The estimates in Table 1 are standard outputs from a data analysis of cross-tables.

The dataset contains three binary variables (A1, A2, and A3) and three polytomous ones (B1, B2, and B3). In the set of A1, A2, and A3, we expect to see PMC = η1 ≠η2 because of (7), and in the set of B1, B2, and B3, we expect to see PMC < η1 because of Eq. (12). Variables A1 and B1 follow a deterministic pattern without tied pairs and without stochastic error and no crossing pairs between *g* and *X*—here, we expect to obtain a perfect association because *X* explains the pattern in *g* in a deterministic manner, although it includes a small number of tied cases to illustrate their effect on the estimates. Variables A2 and B2 include the stochastic error to a minor extent in the response pattern but no crossing pairs between *g* and *X*—here, we expect to see a less than perfect association because of the error in the response pattern, so that PMC = η1 < 1 in A2, PMC < η1 < 1 in B2, and η2 = 1 in both A2 and B2. Variables A3 and B3 include tied pairs, more stochastic errors, and crossing pairs between *g* and *X*—here, we expect to see a less than perfect association and a pattern PMC < η1 < 1 for B3 and η2 < 1 in both A3 and B3. As benchmarks for PMC and η, two measures that can detect the deterministic patterns, *G* and *D*, are shown in the table.

From Table 1, four outcomes are highlighted. First, as expected from Eq. (7), in the binary case with A1–A3, PMC equals η1 but not η2. Second, with polytomous variables B1–B3, PMC is closer to η1 than η2, however, such that PMC < η1, as expected by Eq. (12). Third, of the measures of association in comparison, only *G* and *D* directed so that “*g* given *X*” detect the deterministic pattern in variables A1 and B1 of the measures of association in comparison. Although η2 seemingly detects the deterministic pattern, the value η2 = 1 is obtained only because there are no crossing categories in variables A1, A2, B1, and B2 in relation to *X*. Variables A3 and B3 are constructed so that the category *y*_*i*_ = 6 in *X* is connected with two different categories in *g* (*x*_*i*_ = 0,1) and, hence, η2 ≠ 1. Fourth, unlike *G* and *D*, PMC and η1 cannot indicate the deterministic pattern of association. This is caused by the mismatch in the number of categories of the variables; perfect PMC implies identical scales of the variables, and perfect η implies a dataset without crossing categories. Then, the values η1 = 0.759 and η1 = 0.820 leading to $\eta_{g|X}^{2}=0.576$ and $\eta_{g|X}^{2}=0.672$ reflecting strictly the magnitude of the underestimation of the true association and explaining power.

The direction of PMC can be verified easily by rotating the cross-table data such that *X* is the row factor and *g* is the column factor. The result would be the same as above: PMC is not related to the rows or columns of the cross-table but to the width of the scales of the variables.

### Directionality of product–moment correlation coefficient in the binary and polytomous cases in real-life datasets

**FIGURE 2:**
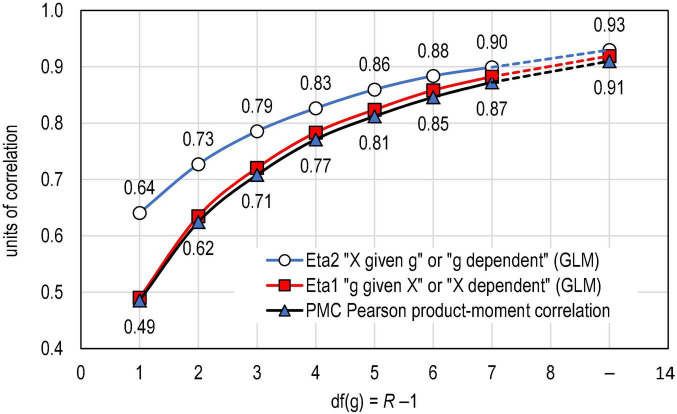
Connection of PMC and eta in real-life datasets (*k* = 11,160 estimates).

Basic statistics related to the empirical dataset discussed in Section “Datasets, methods, and the course of study” are collected in Table 2 and visualised in Figure 2. Notably, the number of items in the dataset decreases by the width of the scale; most of the items are binary (*n* = 5,943), and items with more than 10 categories are sparse.

The empirical dataset supports the idea that PMC is a directional measure not only in the binary case but also when a variable with a narrower scale is polytomous (see Table 2 and Figure 1). This is demonstrated by the fact that when *df*(*g*) > 1, PMC follows closer to η1 than η2. In Figure 1, this is strictly seen in the fact that the curve related to PMC (red line with squares) closely follows the curve related to η1 (blue line with triangles) and not the curve related to η2 (blue line with circles). The difference between PMC and η1 is strictly dependent on how far ${\hat{X}}_{i\,j}$ is from ${\bar{X}}_{j}$, that is, how far the observed dataset is from the linear situation as discussed above.

### How remarkable is the effect of directionality in product–moment correlation coefficient?

In the training dataset, the average difference between PMC and η1 varies between 0.000 and 0.013 units of correlation following an exponential distribution—possibly referring to a cut-normal distribution. The range is somewhat smaller in the cross-validating dataset varying 0.000–0.009. From the viewpoint of interpreting PMC, more important than the difference between η1 and PMC is the difference between η1 and η2; this reflects strictly how far the estimates by PMC are from the symmetric condition.

The empirical datasets suggest that the closer are the number of categories of the variables the less difference there tends to be between η1 and η2 (see also Figure 1). Then, the closer are the number of categories of the variables the more symmetrical interpretation we can make by PMC as well as by the squared PMC, that is, by the explaining power between the variables. On the other hand, the further the scales of two variables are, the less symmetric interpretation we can make from the squared PMC.

Standard GLM is used to study the effect size of the number of categories in *g*, divided into 13 categories (2–13 and 14–15 combined) in explaining the variation in the difference between η1 and η2. In the training dataset, the effect is high [*F*(12, 11,147) = 409.525; $\eta^{2}=\eta_{g|X}^{2}=0.306$, Cohen’s *f* = 0.664] and even higher in the cross-validating dataset with wider scales in *X* [*F*(12, 29,887) = 1415,501; $\eta^{2}=\eta_{g|X}^{2}=0.362$, Cohen’s *f* = 0.753]. The *post hoc* tests with Šidák’s routine (Šidák, 1967) indicate that when the number of categories exceeds seven (*df*(*g*) > 6), the difference between η1 and η2 is no more significant. In the cross-validating dataset, this threshold comes with eight categories (*df*(*g*) > 7). Because of the large number of estimates, the significance is obvious even with minor practical differences. This is re-evaluated from the effect size viewpoint.

Basic statistics related to the difference between η1 and η2 are collected in Table 3 by the number of categories in the items. Notably, in both the training and cross-validating dataset, the difference gets smaller the more categories the items have. The average standard deviation of the difference in the training dataset is 0.093, whereas, in the cross-validating dataset, it is 0.059. Hence, by using Cohen’s *d*, we can roughly estimate that the mean difference between η1 and η2 of the magnitude of 0.03–0.04 is remarkable: $d_{t\,r\,a\,i\,n\,i\,n\,g}=\frac{0.04}{0.093}=0.430$ and Cohen’s $d_{c\,r\,o\,s\,s\,\text{-}\,v\,a\,l\,i\,d\,a\,t\,i\,n\,g}=\frac{0.025}{0.059}=0.424$ showing a medium effect size (Cohen, 1988). Hence, we conclude that from the effect size perspective, the directionality in PMC seems to be remarkable when *g* has less than six or seven categories assuming a notably wider scale in *X*.

An obvious confounding factor in the interpretation of the number of categories in *g* is that the number of categories in *X* is also related to the matter. Hence, the ratio of the number of categories of *g* and *X* (*C*/*R*) is studied further. The statistic *C*/*R* indicates strictly how much longer the scale of *X* is in comparison with the scale of *g*. This ratio appears to explain well the difference between η1 and η2. The connection is somewhat different in the training dataset with a relatively narrow range of *df*(*X*), causing smaller values in *C*/*R* (≤14.00) in comparison with the cross-validating dataset with a wider range in *df*(*X*), causing wider values (<21.50).

**FIGURE 3:**
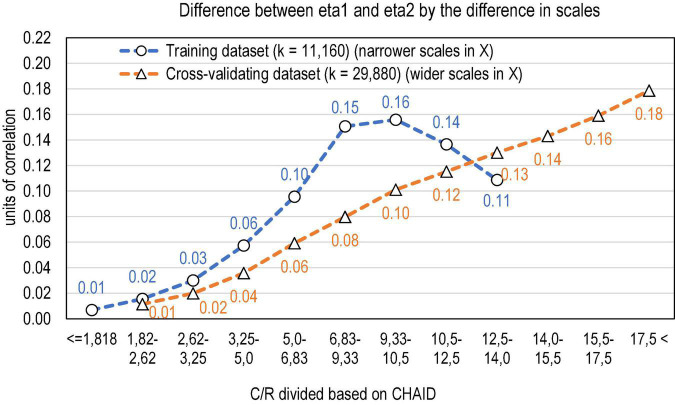
Discrepancy between the scales of the variables explaining the difference in eta1 and eta2 (*k* = 11,160 + 29,887 estimates).

A data mining algorithm CHAID (Kass, 1980; IBM, 2017b) was used in finding the threshold points in the ratio of the number of categories in *g* and *X* that would maximise the significance between the means of η1 and η2. Because of the radically different ranges in the ratio, the datasets were analysed together (*n* = 11,160 + 28,890 estimates) in determining the thresholds. In the combined dataset, CHAID divides the ratio into nine categories from *C*/*R* < 5.0 to *C*/*R* > 17.5 [F(8, 41,038) = 2364,57, *p* < 0.001]. The range was extended by reanalysing the extreme groups without the rest dataset. At the extreme of *C*/*R* < 5, DTA found six detailed groups and, at the extreme of *C*/*R* > 17.5 four detailed groups more, altogether 17 groups. Of these, 11 actualised in the training dataset and 16 in the cross-validating dataset (Table 4 and Figure 3). It is evident that the higher comes *C/R*, that is, the wider is the scale of the score in comparison with the scale of items, the higher gets the discrepancy between η1 and η2 and, consequently, the more directional will be the PMC; the effect sizes are high (Cohen’s *f*_*training*_ = 0.55; *f*_*cross–validating*_ = 0.97).

The association between the ratio of the scales of *X* and *g* and the difference between η1 and η2 is curvilinear in the training dataset at the range of 5 < *C*/*R* < 15 (quadratic *R*^2^ = 0.240 and linear *R*^2^ = 0.182), indicating a magnitude of the correlation that ranges 0.43–0.49. In the cross-validating dataset, the association is clearly more linear (linear *R*^2^ = 0.492), indicating the magnitude of correlation at 0.702. The curvilinearity in the training dataset in comparison with the cross-validating dataset is caused by variety in sample sizes and heteroscedasticity in the phenomenon: the difference between η1 and η2 tends to get wider when the sample size gets lower and the residuals tend to get higher the wider is the difference between the scales of *X* and *g*. In each subsample of *n* = 50, 100, and 200, the linear connection is around the same magnitude as in the cross-validating dataset (linear *R*^2^ = 0.481–0.495). Notably, the higher the sample size gets, the less difference between η1 and η2.

All in all, from the effect size viewpoint, the directionality in PMC tends to be remarkable when *C*/*R*≥4, that is, when the scale of *X* is four times wider than the scale of *g* in measurement modelling settings. In measurement modelling settings with binary items, this means that if the test has more than four (binary) items, PMC is expected to be significantly and remarkably a non-symmetrical measure. Because the datasets are limited to dependent variables g and *X* relevant in measurement modelling settings, the thresholds should be studied further with independent variables before a general conclusion can be drawn about the thresholds in general.

### Relation of product–moment correlation coefficient and *R*_*AC*_

**FIGURE 4:**
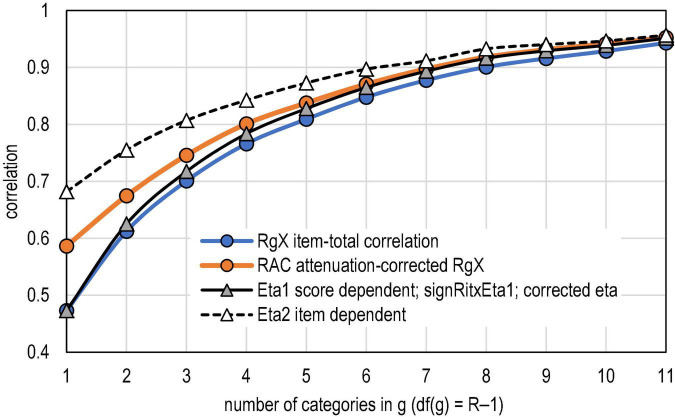
PMC, RAC and coefficient eta.

Finally, a brief empirical note on the relation between PMC and *R*_*AC*_ is given. The estimates by *R*_*AC*_ are always higher or the same as those by PMC. This is inherited from the definition of *R*_*AC*_; when the maximal correlation is reached, then PMC = *R*_*AC*_. Otherwise, PMC < *R*_*AC*_. The relation of these estimators has not been studied, and, hence, it may be generally interesting to know what the effect of the correction is on the PMC. The larger dataset pointed out in text footnote 5 is used in this. Instead of 11,160 estimates as above, an enlarged dataset of 14,880 with a sample size of *n* = 25 is used in this study. Table 5 shows the estimates of correlation by the number of categories in *g*, and Table 6 shows the test statistics for the paired-samples *t*-tests for comparing PMC and *R*_*AC*_ on the one hand, and η1 and *R*_*AC*_ on the other hand. Figure 4 illustrates the differences.

**TABLE 6:** Paired-samples *t*-test of the difference between PMC and RAC and Eta1.

*RgX – RAC*				*Eta1 – RAC*			
df(g)	*t*	*p* (2-sided)	Cohen *d*	*T*	*p* (2-sided)	Cohen *d*	*df*
1	–172.3	<0.001	–1.933	–172.3	<0.001	–1.933	7,947
2	–179.6	<0.001	–3.25	–81.4	<0.001	–1.473	3,055
3	–130.6	<0.001	–3.502	–41.0	<0.001	–1.101	1,389
4	–80.5	<0.001	–2.982	–19.5	<0.001	–0.721	728
5	–56.6	<0.001	–2.601	–9.2	<0.001	–0.423	473
6	–48.9	<0.001	–2.554	–7.0	<0.001	–0.364	365
7	–38.7	<0.001	–2.421	–5.0	<0.001	–0.314	254
8	–32.1	<0.001	–2.712	–2.2	0.027	–0.189	139
9	–25.8	<0.001	–2,036	–2.2	0.028	–0,175	159
10–14	–31.3	<0.001	–1,645	–3.8	<0.001	–0,249	361

The first lift of the relation between PMC and *R*_*AC*_ in the dataset is that the attenuation or deflation in PMC tends to get smaller by the number of categories in *g*. With binary items, the deflation is 19.3% (= (0.586 – 0.473)/0.586 = 0.193), whereas with five categories, the deflation is 4.4%. In both extremes, the difference is statistically significant when the paired-samples *t*-test is used, and the difference is remarkable when evaluated by using Cohen’s *d* (*d* > 1.6; see Table 6). Second, *R*_*AC*_ tends to follow η1 when six categories are reached. This is indicated also by Cohen’s *d*; with *df*(*g*) ≥ 5, — *d*— < 0.43; see Table 6). Notably, the dataset does not include extremely difficult or easy items; and, hence, the deflation appears to be moderate (12.3% on average) instead of being a radical one. With radically more extreme difficulty levels, the deflation is more notable; in the dataset, the highest deflation rate was 67.7%, and in 50 cases, it exceeded 50%. That is, the observed correlation may be 0.36, and this may be the highest possible estimate it can reach. This pattern is typical with items with extreme difficulty levels.

## Discussion and limitations

### Conclusion and discussion

This article reminds, reveals, and studies the hidden characteristics of product–moment coefficient of correlation of being a directional statistic rather than a symmetric one as it is often taken in the textbook materials. The starting point of this article was that PMC is not a symmetric measure of association if the number of categories of variables differs from each other. The research interest in the study was in the effects of the hidden directionality in PMC. This was studied by comparing PMC with coefficient eta. In different types of settings related to the use of correlation coefficients, the interpretations of the results vary.

Within the general settings of *correlational studies*, the explaining power by PMC between a variable with a narrower scale (*g*) and a wider scale (*X*) ($\rho_{g|X}^{2}$) indicates the extent to which *X* explains *g* which, in the language of conditions, can be expressed by using the phrase “association when *g* given *X*.” The deflation related to the pattern that the scale in one variable is notably (more than four times) wider than that in the other variables causes notable deflation also in the explaining power (*r*^2^). Similarly, the explaining power related to the squared multiple correlation (*R*^2^) in the settings related to regression analysis is deflated although its magnitude in the practical settings was not studied in this article. In both cases, the amount of deflation could be assessed by using maximal possible correlation given the dataset or the attenuation-corrected PMC (*R*_*AC*_) or distance correlation as a benchmarking statistics. Systematic studies in this regards would be beneficial.

Within the *settings related to measurement modelling* related to items (*g*) and scores or measurement scales (*X*), PMC and the related explaining power ($\rho_{g|X}^{2}$) are always directed so that the score or measurement scale explains the response pattern in an item—not the other way around or symmetrically. Here, also, the expression related to conditions (“*g* given *X*”) is relevant. This means that the traditional item–total correlation is a directional measure that indicates how well the score explains the response pattern in the item. This makes sense in the measurement modelling settings (e.g., Byrne, 2016; Metsämuuronen, 2017), and, hence, the directional nature of point biserial and point polyserial correlation or item–score correlation can be taken as a positive matter. In these settings, the deflation in the estimates has a notable effect on the negative bias in the estimates of reliability. Metsämuuronen (2022d,e, f) suggested using deflation-corrected estimators of reliability where the deflation-prone estimator or correlation PMC would be replaced by some other better-behaving estimators. Some of these are *R*_*AC*_ and *E*_*AC*_, as well as *D* and *G*, discussed in this article (see Metsämuuronen, 2022f).

Within the *settings related to GLM* with dichotomous independent variables, the direction of PMC equals the traditional direction related to η^2^, and in the polytomous ordinal case, it corresponds closely to the traditional direction of η^2^ directed the way we usually use η^2^ in the GLM settings. In these settings, the opposite naming (“*X* dependent”) is traditionally used ($\rho_{X|g}^{2}$). However, in these settings too, in the case of binary or ordinal settings, *X* explains the *order* of the responses in *g*, and hence with a dichotomous and ordinal *g*, we could call this also “*g* given *X*” or “*g* dependent” ($\rho_{g|X}^{2}$). While eta and eta squared are strictly related to PMC, they are also prone to give notable understimation of association as well as explaining power. Then, the attenuation-corrected eta and attenuation-corrected eta squared ($E_{A\,C}^{2}$) could be used as a benchmark for the magnitude of the probable deflation (see Metsämuuronen, 2022a).

So far, the results are clear. The question is what the consequences of the directionality in PMC are in real-life settings. For many practical settings, the directionality has no effect. For example, if the variables are (essentially) continuous or when the variables have an identical scale, directionality is not a relevant issue. Also, if the scales are near each other and the sample size is high, the directionality seems not to have practical relevance—the issue is merely in principle rather than practical.

However, when there is a radical asymmetry in the scales of two variables, PMC is always a directional measure, and this may have notable practical consequences. This should be kept in mind when estimating the association between a variable with a narrow scale and a variable with a wider metric (ordinal, interval, or continuous) scale, which is more than four times wider than the scale of the other variable. Also, *R*^2^ seen in the standard scatterplots of the bivariate correlation related to PMC or in the ordinary least square regression is not a symmetric but a directional statistic, so that the variable with a wider scale explains the order in the variable with a narrower scale. Deflation in *R*^2^ (or in eta squared) leads us to conclude that the explaining power, traditionally nuanced as “the proportion of remaining variance,” means, factually, “the proportion of remaining variance of which the coefficient *can reach*” (Metsämuuronen, 2022a). Hays (1963, p. 505; see also Richardson, 1996) pointed out the same thing: The proportion of the total variation in the dependent variable is what *can* be predicted or explained based on its regression on the independent variable within the sample being studied. A possible solution is to calculate the maximal PMC, if not *R*_*AC*_, for the given dataset as a benchmark: These indicate how far the observed correlation is from the maximal possible correlation in the given dataset. This reflects the magnitude of deflation. Another relevant benchmark could be the efficiency measure *E* suggested recently by Onyutha (2022); this is based on distance correlation instead of PMC. However, based on Figure 1 and related procedure, it seems that the distance correlation is not specifically strong in the measurement modelling settings at least with binary items. From this perspective, Onyutha’s *E* based on distance correlation may not fully solve the challenge of deflation caused by the discrepancy between the scales of variables. Obviously, systematic studies would be beneficial to understand the boundaries and strengths of these estimators.

It may also be good to rethink the possible consequences of directionality and related deflation in widely used routines for, say, factor analysis, structural equation modelling analysis, and regression analysis, to conclude whether there is something to amend in our practices or the interpretation of the estimates. For the estimation of reliability, several new solutions have been suggested based on changing the PMC in the traditional estimators of reliability by better-behaving estimators; one of these is *R*_*AC*_ (see Metsämuuronen, 2022e,f). At least, it would be good to rethink and rephrase our textbooks and teaching, if needed, of the “symmetricity” related to PMC.

### Limitations and suggestions for further studies

An obvious restriction in the article is that the relation of PMC and η_*g*|*X*_ is derived algebraically only for a binary and dichotomous case. The connection in the polytomous ordinal cases is not as obvious to show as in the binary case (see, however, relevant formulae in Wherry and Taylor, 1946); overall, with the polytomous variables, the case of PMC = η_*g*|*X*_ is a very rare specific case which may not need to be shown in the first place. Nevertheless, the connection is obvious in empirical datasets. Deriving the polytomous ordinal case algebraically may increase our understanding of the matter; polytomous nominal case does not make sense.

Another relevant obvious limitation is related to the datasets used in the empirical part of this article. Although the basic results and derivations in this article are general, practical illustrations of the connection between PMC and η were made by using datasets relevant to measurement modelling settings and specifically item analysis settings. In these settings, two variables, item *g* and score *X*, are mechanically dependent, and they both are reflections of a common latent variable (θ). In these settings, the higher the number of categories gets in the items and the less there are items comprising the test score, the closer the correlation between *g* and *X* approximates the value 1. This phenomenon does not make sense outside the measurement modelling settings. Some relevant threshold values based on the empirical datasets were discussed within the text: how many categories *g* needs to have and how much longer the scale of *X* needs to be in comparison with the scale of *g* to obtain the remarkable effect of directionality in PMC. These thresholds (seven or eight categories and four times longer) may be applicable in measurement modelling settings, although replications of the design and independent studies of the thresholds would confirm or specify the thresholds. This needs to be studied with simulations of truly independent variables.

## Data availability statement

The original contributions presented in this study are included in the article/Supplementary material, further inquiries can be directed to the corresponding author.

## Ethics statement

Ethical review and approval was not required for the study on human participants in accordance with the local legislation and institutional requirements. Written informed consent from the participants’ legal guardian/next of kin was not required to participate in this study in accordance with the national legislation and the institutional requirements.

## Author contributions

The author confirms being the sole contributor of this work and has approved it for publication.

## Footnotes

1 Although usually referred to as the “Pearson correlation,” it was, factually, French physicist Auguste Bravais (1844) who derived the coefficient first. Hence, sometimes the coefficient is called the “Bravais–Pearson correlation” (e.g., Cleff, 2019). See also Stigler (1989) of the relevance of Sir Francis Galton in the development of the coefficient.

2 Metsämuuronen (2022a) reminds us that Kuder (1937) seems to be the first to use the term “point biserial correlation.” He also provided the computational forms. However, Swineford (1936) seems to be the first who studied the binary case of Pearson correlation in relation to biserial correlation (see Moses, 2017).

3 This confusing naming of the directions is discussed in detail by Metsämuuronen (2020a,2022a). In the literature related to directional coefficients (e.g., Siegel and Castellan, 1988; Newson, 2002, 2006; IBM, 2017a; Metsämuuronen, 2017) and, consequently, in the outputs of some generally known software packages, such as IBM SPSS and SAS, as well as *R* libraries, this direction of η is labelled “*X* dependent” and it is notated as η(*X*|*g*). This label makes sense in GLM settings where a test results (*X*) cannot explain the sex (*g*), as an example and, hence, *X* must be “dependent.” An opposite name for the *same* direction is relevant within the measurement modelling settings and non-parametric settings (see discussion related to Table 1 and Metsämuuronen, 2020a). Within the measurement modelling settings, this direction means that the latent trait (θ) manifested as a score variable (*X*) explains the response pattern in the item (*g*), and the opposite direction does not make sense (e.g., Byrne, 2016; Metsämuuronen, 2017). In manual calculations of Mann–Whitney *U* test (Mann and Whitney, 1947) and Jonckheere–Terpstra *JT* test (chronologically, Terpstra, 1952; Jonckheere, 1954), as examples, *X* is first used to order *g* after which the *order* in *g* is analysed. In these settings, it is natural to think that “*g* depends on *X*,” that is, from the condition’s viewpoint, “*g* given *X*,” usually notated as (*g*| *X*). This latter notation η(*g*|*X*) = η_*g*|*X*_ = “eta directed so that ‘*g* given *X’*” is used in the text. The notation η_*g*|*X*_ is preferred over η(*g*|*X*)because it is easy to modify to the form eta squared, $\eta_{g|X}^{2}$.

4 Sincere thanks to Counsellor or Evaluation, Jukka Marjanen from Finnish Education Evaluation Center (FINEEC) for computing the estimate of distance correlation. R package «Energy» by Rizzo and Székely (2022) was used in computing.

5 This somewhat wider training dataset with an additional sample size of *n* = 25, is also published. A dataset of individual items (*n* = 14,880) including relevant indicators of item–score association although without the cross-valiadating dataset is available in CSV format at http://dx.doi.org/10.13140/RG.2.2.10530.76482 and in SPSS format at http://dx.doi.org/10.13140/RG.2.2.17594.72641.

## References

[B1] Ayres L. P. (1920). The Correlation Ratio. *J. Educ. Res.* 2 452–456. 10.1080/00220671.1920.10879073

[B2] Biggs D. de Ville B. Suen E. (1991). A method of choosing multiway partitions for classification and decision trees. *J. Appl. Stat.* 18 49–62. 10.1080/02664769100000005

[B3] Bravais A. (1844). Analyse Mathematique. Sur les probabilités des erreurs de situation d’un point. (Mathematical analysis. Of the probabilities of the point errors). *Mémoires présentés par divers savants à l’Académie Royale des Siences de l’Institut de France (Mem. Present. Various scholars R. Acad. Sci. Institute France)* 9 255–332.

[B4] Breiman L. Friedman J. H. Olshen R. A. Stone C. J. (1984). *Classification and Regression Trees.* Belmont, CA: Wadsworth.

[B5] Byrne B. M. (2016). *Structural Equation Modeling with AMOS. Basic Concepts, Applications, and Programming*, 3rd Edn. London: Routledge.

[B6] Camp B. H. (1933). Karl Pearson and Mathematical Statistics. *J. Am. Stat. Assoc.* 28 395–401. 10.1080/01621459.1933.10503239

[B7] Chan D. (2008). “So why ask me? Are self-report data really that bad?,” in *Statistical and Methodological Myths and Urban Legends*, eds Lance C. E. Vanderberg R. J. (London: Routledge), 309–326. 10.4324/9780203867266

[B8] Chaudhuri A. Hu W. (2019). A fast algorithm for computing distance correlation. *Comput. Stat. Data Anal.* 135 15–24. 10.1016/j.csda.2019.01.016

[B9] Cleff T. (2019). *Applied Statistics and Multivariate Data Analysis for Business and Economics. A Modern Approach Using SPSS, Stata, and Excel.* Berlin: Springer.

[B10] Cohen J. (1969). *Statistical Power Analysis for the Behavioral Sciences.* Cambridge, MA: Academic press.

[B11] Cohen J. (1988). *Statistical Power Analysis for the Behavioral Sciences*, 2nd Edn. Mahwah, NJ: Erlbaum.

[B12] Drasgow F. (1986). “Polychoric and polyserial correlations,” in *Encyclopedia of Statistical Sciences*, Vol. 7 eds Kotz S. Johnson N. L. (New York, NY: John Wiley), 68–74.

[B13] Eikeland H. M. (1971). On the Generality of Univariate Eta. *Scand. J. Educ. Res.* 15 149–167. 10.1080/0031383710150109

[B14] Ekström J. (2011). The Generalized Definition of the Polychoric Correlation Coefficient. UCLA, Department of Statistic Papers. Available online at: https://escholarship.org/uc/item/583610fv (accessed October 7, 2022).

[B15] FINEEC (2018). *National Assessment of Learning Outcomes in Mathematics at Grade 9 in 2002 (Unpublished Dataset Opened for the re-Analysis 18.2.2018).* Helsinki: Finnish Education Evaluation Centre.

[B16] Fisher R. (1925). *Statistical Methods for Research Workers.* Edinburgh: Oliver and Boyd.

[B17] Gadermann A. M. Guhn M. Zumbo B. D. (2012). Estimating ordinal reliability for Likert-type and ordinal item response data: A conceptual, empirical, and practical guide. *Pract. Assess. Res.Eval.* 17 1–13. 10.7275/n560-j767

[B18] Galton F. (1889). Kinship and correlation. *Stat. Sci.* 4, 80–86. 10.1214/ss/1177012581

[B19] Gleeson P. (2018). *Finding Correlations in Non-Linear Data. #Machine Learning. FreeCodeCamp.* Available online at: https://www.freecodecamp.org/news/how-machines-make-predictions-finding-correlations-in-complex-data-dfd9f0d87889/ (accessed October 7, 2022).

[B20] Goodman L. A. Kruskal W. H. (1954). Measures of association for cross classifications. *J. Am. Stat. Assoc.* 49 732–764. 10.1080/01621459.1954.10501231

[B21] Hays W. L. (1963). *Statistics for psychologists.* New York, NY: Holt, Rinehart & Winston.

[B22] Henrysson S. (1963). Correction of item–total correlations in item analysis. *Psychometrika* 28 211–218. 10.1007/BF02289618

[B23] Howell D. G. (2012). *Statistical Methods for Psychology*, 8th Edn. Wadsworth: Duxbury Press.

[B24] IBM (2017a). *IBM SPSS Statistics 25 Algorithms. IBM.* Available online at: https://www.ibm.com/docs/en/SSLVMB_25.0.0/pdf/en/IBM_SPSS_Statistics_Algorithms.pdf (accessed October 7, 2022).

[B25] IBM (2017b). *IBM SPSS Decision Trees 25. IBM.* Available online at: https://www.ibm.com/docs/en/SSLVMB_25.0.0/pdf/en/IBM_SPSS_Decision_Trees.pdf (accessed October 7, 2022).

[B26] Jonckheere A. R. (1954). A distribution-free k–sample test against ordered alternatives. *Biometrika* 41 133–145. 10.1093/biomet/41.1-2.133

[B27] Kass G. (1980). An exploratory technique for investigating large quantities of categorical data. *Appl. Stat.* 29 119–127. 10.2307/2986296

[B28] Kelley T. L. (1935). An unbiased correlation ratio measure. *Proc. Natl. Acad. Sci. U.S.A.* 21 554–559. 16577689 10.1073/pnas.21.9.554 PMC1076656

[B29] Kendall M. (1938). A new measure of rank correlation. *Biometrika* 30 81–93. 10.2307/2332226

[B30] Kendall M. G. (1948). *Rank Correlation Methods*, 1st Edn. Glasgow, GB: Charles Griffin and Co Ltd.

[B31] Kuder G. F. (1937). Nomograph for point biserialr, biserialr, and fourfold correlations. *Psychometrika* 2 135–138. 10.1007/BF02288067

[B32] Kvålseth T. O. (1985). Cautionary note about R2. *Am. Stat.* 39 279–285. 10.1080/00031305.1985.10479448

[B33] Lane S. Raymond M. R. Haladyna T. M. (2016). *Handbook of Test Development*, 2nd Edn. London: Routledge.

[B34] Livingston S. A. Dorans N. J. (2004). *A Graphical Approach to Item Analysis.* (Research Report No. RR-04-10). Princeton, NJ: Educational Testing Service, 10.1002/j.2333-8504.2004.tb01937.x

[B35] Loh W. Y. Shih Y. S. (1997). Split selection methods for classification trees. *Stat. Sinica* 7 815–840.

[B36] Lord F. M. Novick M. R. Birnbaum A. (1968). *Statistical Theories of Mental Test Scores.* Boston: Addison –Wesley Publishing Company.

[B37] Mann H. B. Whitney D. R. (1947). On a test of whether one of two random variables is stochastically larger than the other. *Ann. Math. Stat.* 18 50–60. 10.1214/aoms/1177730491

[B38] Martin W. S. (1973). The effects of scaling on the correlation coefficient: A test of validity. *J. Market. Res.* 10 316–318. 10.2307/3149702

[B39] Martin W. S. (1978). Effects of scaling on the correlation coefficient: Additional considerations. *J. Market. Res.* 15 304–308. 10.1177/002224377801500219

[B40] Meade A. W. (2010). “Restriction of range,” in *Encyclopedia of Research Design*, ed. Salkind N. J. (Thousand Oaks, CA: SAGE Publications, Inc), 1278–1280. 10.4135/9781412961288.n309

[B41] Mendoza J. L. Mumford M. (1987). Corrections for attenuation and range restriction on the predictor. *J. Educ. Stat.* 12 282–293. 10.3102/10769986012003282

[B42] Metsämuuronen J. (2017). *Essentials of Research Methods in Human Sciences*, Vol. 1–3. Thousand Oaks, CA: SAGE Publications, Inc.

[B43] Metsämuuronen J. (2020a). Somers’ D as an alternative for the item–test and item–rest correlation coefficients in the educational measurement settings. *Int. J. Educ. Methodol.* 6 207–221. 10.12973/ijem.6.1.207

[B44] Metsämuuronen J. (2020b). Dimension-corrected Somers’ D for the item analysis settings. *Int. J. Educ. Methodol.* 6 297–317. 10.12973/ijem.6.2.297

[B45] Metsämuuronen J. (2021b). Directional nature of Goodman-Kruskal gamma and some consequences. Identity of Goodman-Kruskal gamma and Somers delta, and their connection to Jonckheere-Terpstra test statistic. *Behaviormetrika* 48 283–307. 10.1007/s41237-021-00138-8

[B46] Metsämuuronen J. (2021a). Goodman–Kruskal gamma and dimension-corrected gamma in educational measurement settings. *Int. J. Educ.Methodol.* 7 95–118. 10.12973/ijem.7.1.95

[B47] Metsämuuronen J. (2022a). Artificial systematic attenuation in eta squared and some related consequences. Attenuation-corrected eta and eta squared, negative values of eta, and their relation to Pearson correlation. *Behaviormetrika.* 10.1007/s41237-022-00162-2 [Epub ahead of print].

[B48] Metsämuuronen J. (2022b). Reminder of the directional nature of the product–moment correlation coefficient. *Academia Lett.* 5313. 10.20935/AL5313 PMC9619107 36324778

[B49] Metsämuuronen J. (2022d). Effect of various simultaneous sources of mechanical error in the estimators of correlation causing deflation in reliability. Seeking the best options of correlation for deflation-corrected reliability. *Behaviormetrika* 49 91–130. 10.1007/s41237-022-00158-y

[B50] Metsämuuronen J. (2022c). Rank–polyserial correlation: Quest for a “missing” coefficient of correlation. *Front. Appl. Math. Stat.* 8:914932. 10.3389/fams.2022.914932

[B51] Metsämuuronen J. (2022e). Attenuation-corrected reliability and some other MEC-corrected estimators of reliability. *Appl. Psychol. Measur.* 10.1177/01466216221108131 [Epub ahead of print]. PMC9574086 36262520

[B52] Metsämuuronen J. (2022f). Typology of deflation-corrected estimators of reliability. *Front. Psychol.* 13:891959. 10.3389/fpsyg.2022.891959 35923730 PMC9341485

[B53] Mordkoff J. T. (2019). A simple method for removing bias from a popular measure of standardized effect size: Adjusted partial eta squared. *Adv. Methods Practices Psychol. Sci.* 2 228–232. 10.1177/2515245919855053

[B54] Moses T. (2017). “A review of developments and applications in item analysis,” in *Advancing Human Assessment. The Methodological, Psychological and Policy Contributions of ETS*, eds Bennett R. von Davier M. (London: Springer Open), 19–46. 10.1007/978-3-319-58689-2_2

[B55] Newson R. (2002). Parameters behind “nonparametric” statistics: Kendall’s tau, Somers’ D and median differences. *Stata J.* 2 45–64.

[B56] Newson R. (2006). Confidence intervals for rank statistics: Somers’ D and extensions. *Stata J.* 6 309–334.

[B57] Okada K. (2013). Is omega squared less biased? A comparison of three major effect size indices in one-way ANOVA. *Behaviormetrika* 40 129–147. 10.2333/bhmk.40.129

[B58] Okada K. (2017). Negative estimate of variance-accounted-for effect size: How often it is obtained, and what happens if it is treated as zero. *Behav. Res. Methods* 49 979–987. 10.3758/s13428-016-0760-y 27334468

[B59] Olsson U. (1980). Measuring Correlation in Ordered Two-Way Contingency Tables. *J. Market. Res.* 17 391–394. 10.1177/002224378001700315

[B60] Olsson U. Drasgow F. Dorans N. J. (1982). The polyserial correlation coefficient. *Psychometrika* 47 337–347. 10.1007/BF02294164

[B61] Onyutha C. (2022). A hydrological model skill score and revised R-squared. *Hydrol. Res.* 53 51–64. 10.2166/nh.2021.071

[B62] Pearson K. (1896). VII. Mathematical contributions to the theory of evolution. III. Regression, heredity and panmixia. *Philosophical. Trans. R. Soc. London* 187 253–318. 10.1098/rsta.1896.0007

[B63] Pearson K. (1900). I. Mathematical contributions to the theory of evolution. VII. On the correlation of characters not quantitatively measurable. Philosophical Transactions of the Royal Society A. *Math. Physical. Eng. Sci.* 195 1–47. 10.1098/rsta.1900.0022

[B64] Pearson K. (1903). I. Mathematical contributions to the theory of evolution. XI. On the influence of natural selection on the variability and correlation of organs. Philosophical Transactions of the Royal Society A. *Math. Physical. Eng. Sci.* 200 1–66. 10.1098/rsta.1903.0001

[B65] Pearson K. (1904). *On the Theory of Contingency and its Relation to Association and Normal Correlation. Drapers’ Company Research Memoirs. Biometric Series I, XIII. Dulau and Co.* Available online at: http://archive.org/details/cu31924003064833 (Accessed October 7, 2022).

[B66] Pearson K. (1905). *On the General Theory of Skew Correlation and Non-Linear Regression. Dulau and Co.* Available online at: https://archive.org/details/ongeneraltheory00peargoog/page/n3 (Accessed October 7, 2022).

[B67] Pearson K. (1911). On a correction to be made to the correlation ratio η. *Biometrika* 8 254–256. 10.2307/2331454

[B68] Pearson K. (1913). On the Measurement of the Influence of “Broad Categories” on Correlation. *Biometrika* 9 116–139. 10.1093/biomet/9.1-2.116

[B69] Richardson J. T. E. (1996). Measures of effect size. *Behav. Res. Methods Instrum. Comput.* 28, 12–22. 10.3758/BF03203631

[B70] Rizzo M. Székely G. (2022). *E-Statistics: Multivariate Inference via the Energy of Data.* Available online at: https://cran.r-project.org/web/packages/energy/index.html (Accessed October 7, 2022).

[B71] Sackett P. R. Lievens F. Berry C. M. Landers R. N. (2007). A cautionary note on the effect of range restriction on predictor intercorrelations. *J. Appl. Psychol.* 92 538–544. 10.1037/0021-9010.92.2.538 17371098

[B72] Sackett P. R. Yang H. (2000). Correction for range restriction: An expanded typology. *J. Appl. Psychol.* 85 112–118. 10.1037/0021-9010.85.1.112 10740961

[B73] Salkind N. J. (ed.) (2010). *Encyclopedia of Research Design.* London: SAGE Publications, Inc.

[B74] Schmidt F. L. Hunter J. E. (2003). “History, development, evolution, and impact of validity generalization and meta-analysis methods, 1975–2001,” in *Validity Generalization: A Critical Review*, ed. Murphy K. R. (Mahwah, NJ: Erlbaum), 31–66.

[B75] Schmidt F. L. Hunter J. E. (2015). *Methods of Meta-Analysis: Correcting Error and Bias in Research Findings*, 3rd Edn. London: SAGE Publications, Inc, 10.4135/9781483398105

[B76] Schmidt F. L. Shaffer J. A. Oh I.-S. (2008). Increased accuracy for range restriction corrections: Implications for the role of personality and general mental ability in job and training performance. *Personnel Psychol.* 61 827–868. 10.1111/j.1744-6570.2008.00132.x

[B77] Sechrest L. Yeaton W. H. (2011). “Magnitudes of experimental effects in social science research,” in *SAGE Directions of Educational Psychology*, Vol. IV, ed. Salkind N. J. (London: SAGE Publications Inc), 3–22.

[B78] Šidák Z. (1967). Rectangular confidence region for the means of multivariate normal distributions. *J. Am. Stat. Assoc.* 62 626–633. 10.1080/01621459.1967.10482935

[B79] Siegel S. Castellan N. J. Jr. (1988). *Nonparametric Statistics for the Behavioural Sciences*, 2nd Edn. New York, NY: McGraw-Hill.

[B80] Silver N. C. (2008). “Attenuation,” in *Encyclopedia of Survey Methods*, ed. Lavrakas P. J. (London: Sage Publications, Inc), 37–37.

[B81] Somers R. H. (1962). A new asymmetric measure of association for ordinal variables. *Am. Soc. Rev.* 27 799–811. 10.2307/2090408

[B82] Spearman C. (1904). The proof and measurement of association between two things. *Am. J. Psychol.* 15 72–101. 10.2307/1422689 3322052

[B83] Stigler S. M. (1989). Francis Galton’s account of the invention of correlation. *Stat. Sci.* 4 73–79. 10.1214/ss/1177012580

[B84] Swineford F. (1936). Biserial r versus Pearson r as measures of test-item validity. *J. Educ. Psychol.* 27 471–472. 10.1037/h0052118

[B85] Székely G. J. Rizzo M. L. Bakirov N. K. (2007). Measuring and testing dependence by correlation of distances. *Ann. Stat.* 35 2769–2794. 10.1214/009053607000000505

[B86] Tabachnick B. G. Fidell L. S. (2013). *Using Multivariate Statistics*, 6th Edn. London: Pearson Education.

[B87] Terpstra T. J. (1952). The asymptotic normality and consistency of Kendall’s test against trend, when ties are present in one ranking. *Indagationes Math.* 14 327–333. 10.1016/S1385-7258(52)50043-X

[B88] Walk M. J. Rupp A. A. (2010). “Pearson product-moment correlation coefficient,” in *Encyclopedia of Research Design*, ed. Salkind N. J. (London: SAGE Publications, Inc), 1022–1026.

[B89] Wherry R. J. Taylor E. K. (1946). The relation of multiserial eta to other measures of correlation. *Psychometrika* 11 155–161. 10.1007/BF02289296 20288937

[B90] Yang H. (2010). “Factor loadings,” in *Encyclopedia of Research Design*, ed. Salkind N. J. (London: SAGE Publications, Inc), 480–483.

