# Supplementary material for: Directional nature of the product–moment correlation coefficient and some consequences
Source: Front Psychol. 2022 Oct 17;13:988660. doi: 10.3389/fpsyg.2022.988660 (PMC9619107; doi:10.3389/fpsyg.2022.988660)
Supplement: Supplementary file 1 [file Data_Sheet_1.PDF]

Appendix 1. Algebraic reasons why PMC underestimates association with a binary *g* and a metric *X*

Assume a binary variable *g* with observed values or subpopulations $x_i = 0,1$ and a metric variable *X* with observed values y_j. PMC can be expressed in a form

$$PMC = \frac{\left(\bar{X}_{X1} - \bar{X}_{X0}\right) \times \sigma_g}{\sigma_X} \tag{1}$$

where σ_g and σ_X are standard deviations of *g* and *X* and $\bar{X}_{X0}$ and $\bar{X}_{X1}$ refer to the means of the variable *X* in the subpopulations 0,1. Let us denote the grand mean in *X* by GM_X. Then, the variance of *X* can be manipulated as follows:

$$\begin{aligned}
\sigma_X^2 &= \frac{1}{N}\left(\sum_{j=1}^{n_0} (y_j - GM_X)^2 + \sum_{j=1}^{n_1} (y_j - GM_X)^2 \right) \\
&= \frac{1}{N}\left(\sum_{j=1}^{n_0} (y_j - \bar{X}_{X0})^2 + n_0(\bar{X}_{X0} - GM_X)^2 + \sum_{j=1}^{n_1} (y_j - \bar{X}_{X1})^2 + n_1(\bar{X}_{X1} - GM_X)^2 \right) \\
&= \frac{1}{N}\left(n_0 \sum_{j=1}^{n_0} \frac{(y_i - \bar{X}_{X0})^2}{n_0} + n_1 \sum_{j=1}^{n_1} \frac{(y_j - \bar{X}_{X1})^2}{n_1} + n_0(\bar{X}_{X0} - GM_X)^2 + n_1(\bar{X}_{X1} - GM_X)^2 \right) \\
&= \frac{1}{N}\left(n_0\sigma_{X0}^2 + n_1\sigma_{X1}^2 + n_0(\bar{X}_{X0} - GM_X)^2 + n_1(\bar{X}_{X1} - GM_X)^2 \right) \\
&= (1-p)\sigma_{X0}^2 + p\sigma_{X1}^2 + (1-p)(\bar{X}_{X0} - GM_X)^2 + p\left(\bar{X}_{X1} - GM_X\right)^2 .
\end{aligned} \tag{2}$$

The term $\left(\bar{X}_{X1} - \bar{X}_{X0}\right) \times \sigma_g$ can be expressed as follows:

$$\left(\bar{X}_{X1} - \bar{X}_{X0}\right) \times \sigma_g = \left(\left(\bar{X}_{X1} - GM_X\right) - \left(\bar{X}_{X0} - GM_X\right)\right) \times \sigma_g \tag{3}$$

Then, PMC can be expressed in a form

$$PMC = \frac{\left(\left(\bar{X}_{X1} - GM_X\right) - \left(\bar{X}_{X0} - GM_X\right)\right) \times \sigma_g}{\sqrt{p\sigma_{X1}^2 + (1-p)\sigma_{X0}^2 + p(\bar{X}_{X1} - GM_X)^2 + (1-p)(\bar{X}_{X0} - GM_X)^2}} . \tag{4}$$

In the binary case,

$$p\left(\bar{X}_{X1} - GM_X\right) = (1-p)\left(GM_X - \bar{X}_{X0}\right) = -(1-p)\left(\bar{X}_{X0} - GM_X\right) . \tag{5}$$

Then,

$$\left(\bar{X}_{X0} - GM_X\right) = -\frac{p}{(1-p)}\left(\bar{X}_{X1} - GM_X\right) \tag{6}$$

and

$$\left(\bar{X}_{X0} - GM_X\right)^2 = \frac{p^2}{\left(1-p\right)^2}\left(\bar{X}_{X1} - GM_X\right)^2 . \tag{7}$$

Because $\sigma_g = \sqrt{p\left(1-p\right)}$ and because of Eqs. (5), (6), and (7),

Directional nature of PMC

$$PMC = \frac{\left(\left(\bar{X}_{X1} - GM_X\right) + \frac{p}{(1-p)}\left(\bar{X}_{X1} - GM_X\right)\right) \times \sqrt{p(1-p)}}{\sqrt{p\sigma_{X1}^2 + (1-p)\sigma_{X0}^2 + p(\bar{X}_{X1} - GM_X)^2 + \frac{(1-p)p^2}{(1-p)^2}\left(\bar{X}_{X1} - GM_X\right)^2}}$$

$$= \frac{\sqrt{\frac{p}{(1-p)}}\left(\bar{X}_{X1} - GM_X\right)}{\sqrt{\frac{p}{(1-p)}} \times \sqrt{(1-p)\sigma_{X1}^2 + (1-p)\left(\frac{1}{p} - 1\right)\sigma_{X0}^2 + \left(\bar{X}_{X1} - GM_X\right)^2}}$$

$$= \frac{\left(\bar{X}_{X1} - GM_X\right)}{\sqrt{(1-p)\sigma_{X1}^2 + (1-p)\left(\frac{1}{p} - 1\right)\sigma_{X0}^2 + \left(\bar{X}_{X1} - GM_X\right)^2}} \quad . \qquad (8)$$

Strictly from Eq. (8) it is known that $\eta\left(g|X\right) = \eta_{g|X}^2 = 1$ is possible only in the theoretical case of σ_{X1}^2 = σ_{X0}^2 = 0. This conditions implies that both *g* and *X* need to have equal number of categories; if the scale in *X* is wider than that of *g*, $\sigma_{X1}^2, \sigma_{X0}^2 \neq 0$. The highest magnitude in Eq. (8) is achieved when σ_g is the highest, that is, when $p = 0.5$:

$$PMC^{Max} = \frac{\left(\bar{X}_{X1} - GM_X\right)}{\sqrt{0.5 \times \left(\sigma_{X1}^2 + \sigma_{X0}^2\right) + \left(\bar{X}_{X1} - GM_X\right)^2}} \quad . \qquad (9)$$

Assuming symmetric distribution of *X*, the symmetricity with two means determinates that $\sigma_{X1}^2 = \sigma_{X0}^2$. Then, when $p = 0.5$, the maximum magnitude of PMC is

$$PMC^{Max} = \frac{\left(\bar{X}_{X1} - GM_X\right)}{\sqrt{\sigma_{X1}^2 + \left(\bar{X}_{X1} - GM_X\right)^2}} = \frac{\left(\bar{X}_{X0} - GM_X\right)}{\sqrt{\sigma_{X0}^2 + \left(\bar{X}_{X0} - GM_X\right)^2}} \quad . \qquad (10)$$

If the distribution of *X* is even without ties, implying symmetricity, PMC^{Max} approximates 0.866 when the number of cases increases. The value depends on, to some extent, the number of cases. For example, with 10, 20, 30, 200, and 1000 cases, the value is 0.8704, 0.8671, 0.8665, 0.8660, and 0.8660, respectively. These are easy to verify by forming the datasets.

Appendix 2. Identity of PMC and eta in the binary datasets

Assume a binary g with the observed values x_i = 0, 1 and a metric variable X with observed values y_i. Because of Eq. (2) and because $\sigma_g^2 = (1-p)\,p = \left((1-p)+p\right)\times(1-p)\,p = (1-p)\,p^2 + p(1-p)^2$,

$$PMC^2 = \left(\frac{\left(\bar{X}_{X1}-\bar{X}_{X0}\right)\times\sigma_g}{\sigma_X}\right)^2 = \frac{\left(\bar{X}_{X1}-\bar{X}_{X0}\right)^2\times\left[p(1-p)\right]}{\sigma_X^2}$$

$$= \frac{\left(\bar{X}_{X1}-\bar{X}_{X0}\right)^2\left[(1-p)\,p^2 + p(1-p)^2\right]}{\sigma_X^2}$$

$$= \frac{(1-p)\,p^2\left(\bar{X}_{X1}-\bar{X}_{X0}\right)^2 + p(1-p)^2\left(\bar{X}_{X1}-\bar{X}_{X0}\right)^2}{\sigma_X^2}$$

$$= \frac{(1-p)\left(p\bar{X}_{X1}-p\bar{X}_{X0}+\bar{X}_{X0}-\bar{X}_{X0}\right)^2 + p\left((1-p)\,\bar{X}_{X1}-(1-p)\,\bar{X}_{X0}\right)^2}{\sigma_X^2}$$

$$= \frac{(1-p)\left(\bar{X}_{X0}-\left((1-p)\,\bar{X}_{X0}+p\bar{X}_{X1}\right)\right)^2 + p\left(\bar{X}_{X1}-\left((1-p)\,\bar{X}_{X0}+p\bar{X}_{X1}\right)\right)^2}{\sigma_X^2} \qquad (1)$$

The variance of X is

$$\sigma_X^2 = \frac{1}{N}\sum_{i=1}^{N}\left(y_i - GM_X\right)^2 \qquad (2)$$

and, in the binary case, the grand mean on X (GM_X) can be expressed as

$$GM_X = (1-p)\,\bar{X}_{X0} + p\bar{X}_{X1}. \qquad (3)$$

Then, because of Eqs. (1), (2), and (3),

$$PMC^2 = \frac{(1-p)\left(\bar{X}_{X0}-GM_X\right)^2 + p\left(\bar{X}_{X1}-GM_X\right)^2}{\sigma_X^2}\Big|\times N$$

$$= \frac{n_0\left(\bar{X}_{X0}-GM_X\right)^2 + n_1\left(\bar{X}_{X1}-GM_X\right)^2}{\sum_{i=1}^{N}\left(y_i-GM_X\right)^2}, \qquad (4)$$

leading to

$$PMC = \sqrt{\frac{n_0\left(\bar{X}_{X0}-GM_X\right)^2 + n_1\left(\bar{X}_{X1}-GM_X\right)^2}{\sum_{i=1}^{N}\left(y_i-GM_X\right)^2}}. \qquad (5)$$

In the binary case, eta directed so that "g given X" can be expressed as

$$\eta_{g|X} = \sqrt{\frac{n_0\left(\bar{X}_{X0}-GM_X\right)^2 + n_1\left(\bar{X}_{X1}-GM_X\right)^2}{\sum_{i=1}^{N}\left(y_i-GM_X\right)^2}}, \qquad (6)$$

where $\bar{X}_{X0}$ and $\bar{X}_{X0}$ are the means of X in the subpopulations of g = 0,1 and the opposite direction "g dependent" or "X given g" can be expressed as

Directional nature of PMC

$$\eta_{X|g} = \sqrt{\frac{\sum_{X=1}^{C} n_X \left(\bar{g}_{gX} - GM_g\right)^2}{\sum_{i=1}^{N} \left(x_i - GM_g\right)^2}} \quad . \tag{7}$$

where $\bar{g}_{gX}$ refers to the means of g in each category X and GM_g is the grand mean of g. Except the special case that the variables have equal scales with no crossing categories, $\eta_{g|X} \neq \eta_{X|g}$. Then, because of Eqs. (7), (8), and (6), in the binary case,

$$PMC = \eta_{g|X} \neq \eta_{X|g} , \tag{8}$$

that is, PMC is unambiguously a directional measure to only one direction of eta, and this direction is the same direction as we usually use the eta squared in the settings related to general linear modeling. This direction is meaningful from the measurement modelling settings where the score variable (X) explains the behavior or response pattern in the items (g) (see discussion of the directions in 2022a).

Appendix 3. Forming the datasets for the empirical section

Ten random samples of n = 50, 100, and 200 test-takers were picked from the original dataset. In each 10×3 datasets, 36 shorter tests with 372 items were produced by varying the number and the difficulty levels of the items. The tests and the related test items were formed by different compilations of single binary items on the one hand and sub-scores constructed by different item compilations, leading to polytomous items, on the other. As the outcome of the process, a set of 1,080 real-world tests were produced with a different number of test-takers (n = 50, 100, and 200), number of items (k = 2–30, $\bar{k} = 10.22$), difficulty levels ($\bar{p}$ = 0.55–0.76, $\bar{\bar{p}} = 0.661$), reliabilities (α = 0.739–0.935, $\bar{\alpha} = 0.862$), and degrees of freedom in the item ($df(g)$ = R–1 = 1–15, $\overline{df(g)} = 5.06$) and in the score ($df(X)$ = C–1 = 12–27, $\overline{df(X)} = 19.2$). These tests produced 11,160 test items with varying difficulty levels, item variances, number of categories, as well as estimates by PMC and η (see Table 2). This dataset is called the "training dataset".

The training dataset was limited to relative short tests ($df(X)$ < 28). Hence, another dataset, partly artificial, was prepared. This is called "cross-validation dataset" and it was based on the same original dataset as the training dataset such that the original 30 items were doubled with small changes in the order and response patterns of the real test-takers. This had a small effect on the item difficulties and item–total correlations. The original items and the modified ones were combined as a dataset with 60 binary items with a nature of odd–even parallel tests.

The parallel way as in the training dataset, 19 sets of n = 200 test-takers were picked from the randomized original dataset and the tests and test items were formed by using different compilations of single binary items and sub-scores constructed by different item compilations to produce polytomous items. Only tests with the number of items of k = 30, 35, 40, 45, 50, 55, and 60 were produced. Altogether 19 × 72 = 1,368 tests with k = 29,887 items were produced. In this dataset, $df(X)$ varies as 18–42 with the average $\overline{df(X)}$ = 30.4. Because of the process of forming the dataset, the variability in items is remarkably smaller than if genuinely independent 60 items would have been used as a basis for the polytomous items. Hence, the dataset was not used as the primary training dataset.

Appendix 4. List of abbreviations used in the article

General abbreviations	
$df(g)$	degrees of freedom of item = number of categories–1
$df(X)$	degrees of freedom of the score = number of categories–1
g	variable with a narrower scale; observed item
k	number of items
p	probability, proportion of correct answers
X	variable with a wider scale; observed score variable
R	number of categories in a variable with a narrower scale
C	number of categories in a variable with a wider scale
σ_X^2 and σ_Y^2	population variance of variables X and Y
σ_X and σ_Y	standard deviation of variables X and Y
σ_{XY}	population covariance between variables X and Y
σ_{XY}^{Max}	maximal population covariance between variables X and Y
Abbreviations related to estimators of correlation	
$D = D(g\|X)$	Somers delta directed so that "g given X" or "X dependent"
$G = G(g\|X)$	Goodman–Kruskal gamma hiddenly directed so that "g given X" or "X dependent"
E_{AC}	attenuation-corrected eta
$eta = \eta$	coefficient eta
$\eta1 = \eta(g\|X) = \eta_{g\|X}$	eta directed so that "g given X" or "X dependent"
$\eta2 = \eta(X\|g) = \eta_{X\|g}$	eta directed so that "X given g" or "g dependent"
$\eta_{g\|X}^{Max}$ and $\eta_{g\|X}^{Obs}$	maximal and observed coefficient eta between variables X and g
PMC = ρ_{XY}	product-moment correlation coefficient between variables X and Y, «Pearson correlation»
$Rit = \rho_{gX} = \rho_{PB}$	item–total correlation, item–score correlation, point-biserial correlation, a special case of PMC
ρ_{XY}^{Max} and ρ_{XY}^{Obs}	maximal and observed correlation between variables X and Y
R_{AC}	attenuation-corrected PMC
Abbreviations related to explaining power and effect size	
d	Cohen's d
ε^2	epsilon squared
$\eta^2 = \eta_{g\|X}^2$	eta squared
f	Cohen's f
R^2	squared multiple correlation coefficient related to regression analysis
$r^2 = \rho_{g\|X}^2$	r squared, squared correlation coefficient
ω^2	omega squared
abbreviations related to research methods	
CHAID	Chi-square Automatic Interaction Detector, an algorithm in DTA
CRT = CART	*Classification and Regression Trees*, an algorithm in DTA
DTA	decision tree analysis
GLM	general linear modeling

Directional nature of PMC

QUEST	*Quick, Unbiased, Efficient Statistical Tree*, an algorithm in DTA
SS_{total}	total sum of squares
$SS_{residual}$	residual sum of squares
SS_{error}	error sum of squares
$\hat{X}$	predicted value
$\bar{X}$	keskiarvo
